# Supplementary material for: Lithocholic Acid Hydroxyamide Destabilizes Cyclin D1 and Induces G0/G1 Arrest by Inhibiting Deubiquitinase USP2a
Source: Cell Chem Biol. 2017 Apr 20;24(4):458–470.e18. doi: 10.1016/j.chembiol.2017.03.002 (PMC5404848; doi:10.1016/j.chembiol.2017.03.002)
Supplement: Document S1. Figures S1–S5 and Table S1 [file mmc1.pdf]

Cell Chemical Biology, Volume *24*

Supplemental Information

Lithocholic Acid Hydroxyamide Destabilizes Cyclin D1 and Induces G_0/G_1 Arrest by Inhibiting Deubiquitinase USP2a

Katarzyna Magiera, Marcin Tomala, Katarzyna Kubica, Virginia De Cesare, Matthias Trost, Bartosz J. Zieba, Neli Kachamakova-Trojanowska, Marcin Les, Grzegorz Dubin, Tad A. Holak, and Lukasz Skalniak

Supplemental Figures

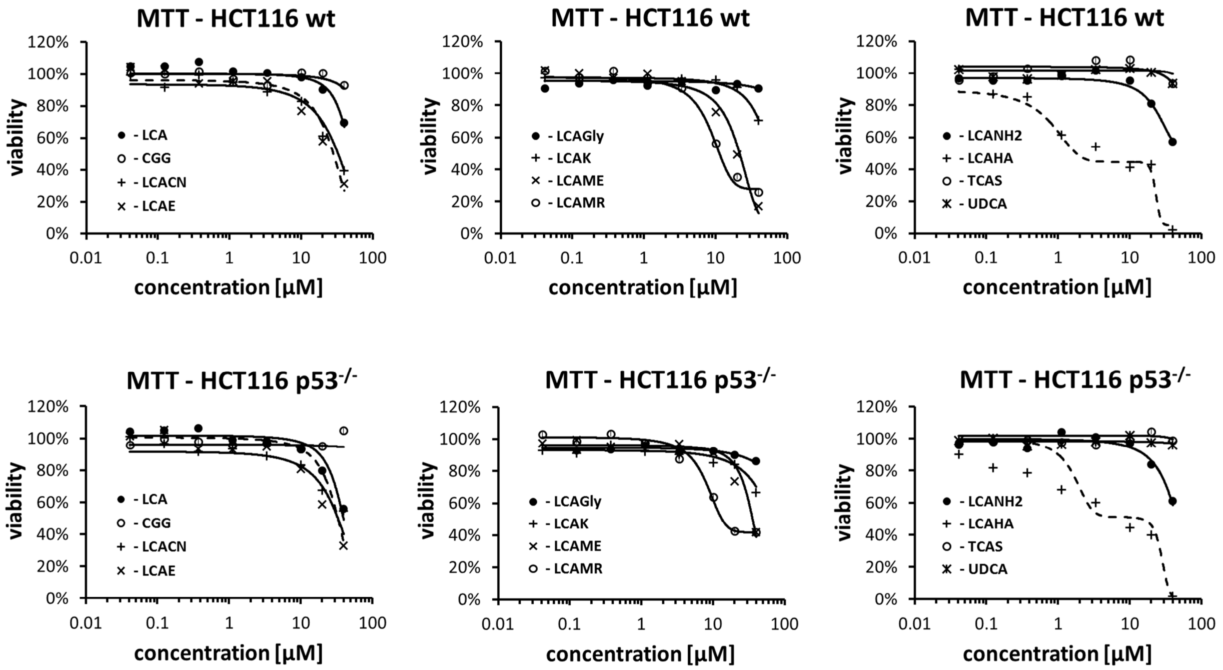

Figure S1. The impact of LCA and its derivatives on the growth and survival of HCT116 p53^{wt} and HCT116 p53$^{-/-}$ cell lines. Related to Figure 1 and Table 2.

For MTT assay, the cells were seeded at low confluence and treated with indicated concentrations of the compounds for six days. The graphs show representative results from three experiments.

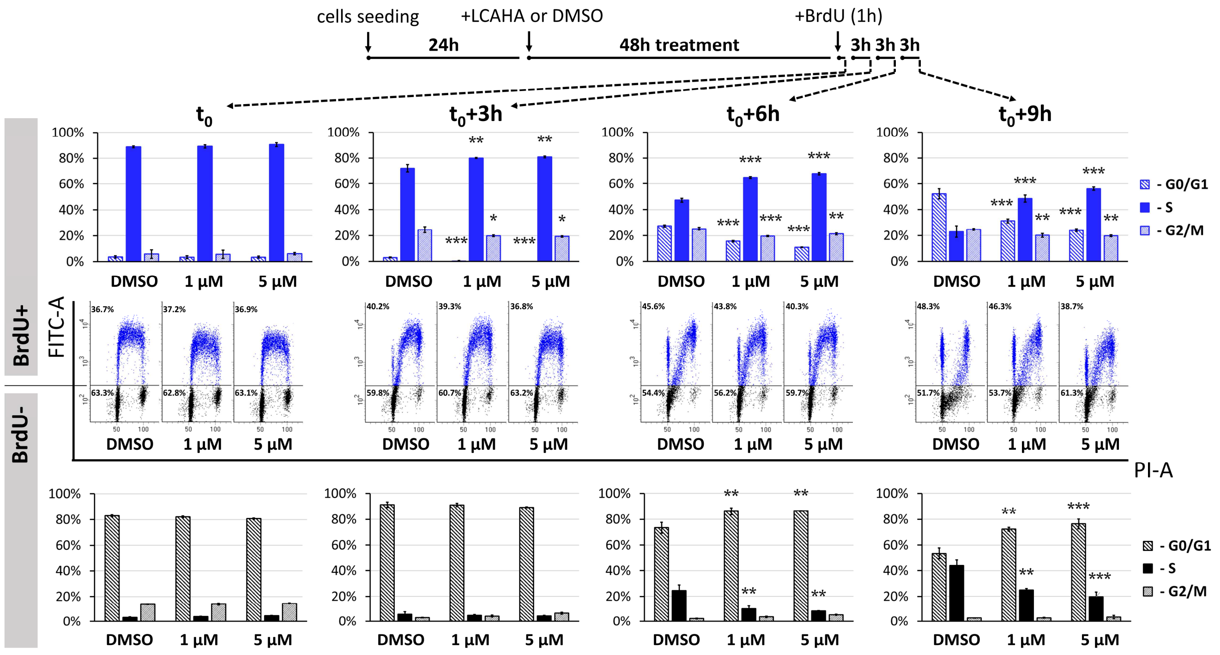

Figure S2. PI-BrdU pulse-chase labeling of HCT116 cells for the evaluation of cell cycle progression following LCAHA treatment. Related to Figure 1.

HCT116 p53^{wt} cells were seeded on culture dishes in growth medium containing 3.3% FBS and treated with LCAHA in a concentration of 1 µM or 5 µM for 48 hours, or with DMSO as a control. One hour before the end of the treatment half of the medium was collected in a falcon tube, and the other half was supplemented with BrdU in a final concentration of 10 µM. After one hour, the medium containing BrdU was removed and the cells were trypsinized and fixed (time “t_0”) or cultured in the medium previously collected in the falcon tube for additional 3, 6 or 9 hours. After that time the cells were trypsinized, fixed and stained with FITC conjugated anti-BrdU antibody and propidium iodide (PI). The cells were analyzed by flow cytometry using BD Fortessa cell analyzer and BD FACSDIVA software. All cells, as well as FITC positive (BrdU+, top panel) and FITC negative (BrdU-, bottom panel) counts were exported to separate files and analyzed separately using ModFit LT Software. The graphs show mean ± SD values from three independent experiments. The statistical significance was evaluated using one-way ANOVA with Tukey’s post-hoc test: * $p<0.05$, ** $p<0.01$, *** $p<0.001$.

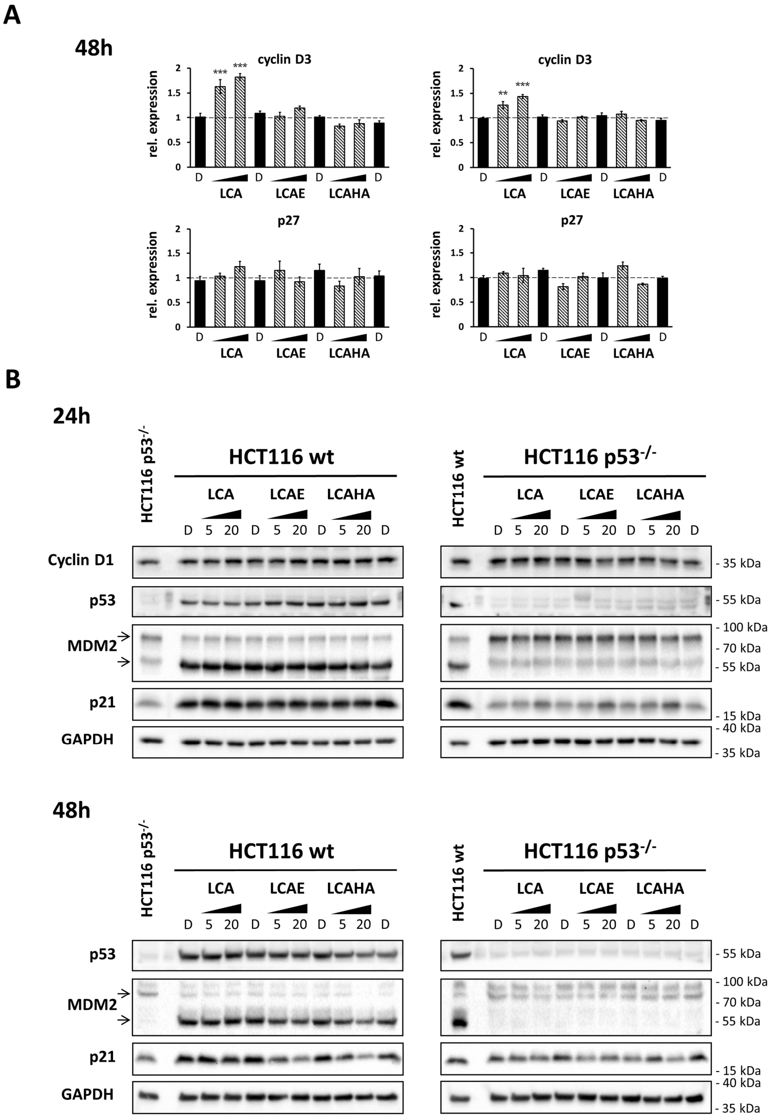

Figure S3. The analysis of the expression of cyclin D3 and p27 and the activation of p53 pathway by LCA, LCAE and LCAHA. Related to Figure 2.

(A) The graphs present densitometry analysis of cyclin D3 and p27 expression form western blot results presented in the Figure 2A of the manuscript. The graphs show mean ± SEM from three independent experiments. The statistical significance was evaluated using one-way ANOVA with Tukey's post-hoc test: ** p<0.01, *** p<0.001.

(B) HCT116 $p53^{wt}$ (left panels) or HCT116 $p53^{-/-}$ (right panels) cells were treated with the indicated concentrations of the compounds for 24 (top) or 48 (bottom) hours. Western blot analysis was performed with the indicated antibodies. The figure shows western blot images representative of three independent experiments.

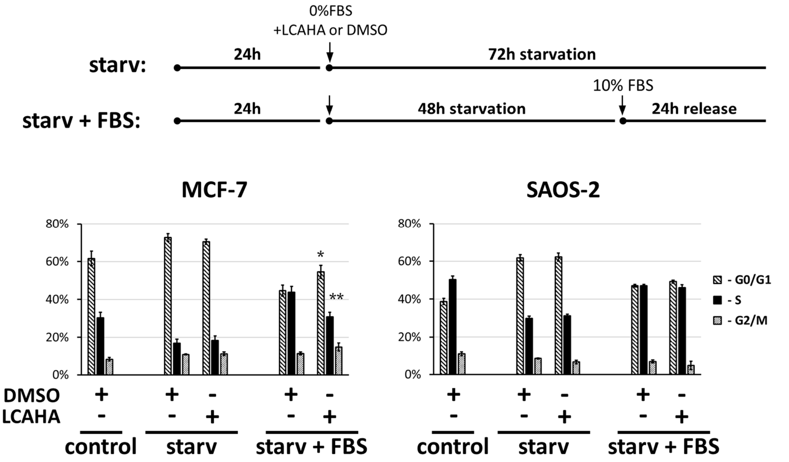

Figure S4. The analysis of the progression of MCF-7 and SAOS-2 cells through the cell cycle following serum starvation and release by the addition of serum. Related to Figure 3.

For the experiment, cyclin D1-dependent MCF-7 and cyclin D1-negative SAOS-2 cell lines were used. The cells were synchronized in G0/G1 by the removal of FBS for 2 days of the culture in the presence of LCAHA compound. After this time, the cells were released by the addition of the medium supplemented with 10% FBS for 24 hours (still in the presence of the compound). The cells were fixed, stained with PI and analysed for cell cycle distribution with ModFit LT Software. The graphs show mean ± SD values from three independent experiments. The statistical significance was evaluated using t-test: * $p<0.05$, ** $p<0.01$.

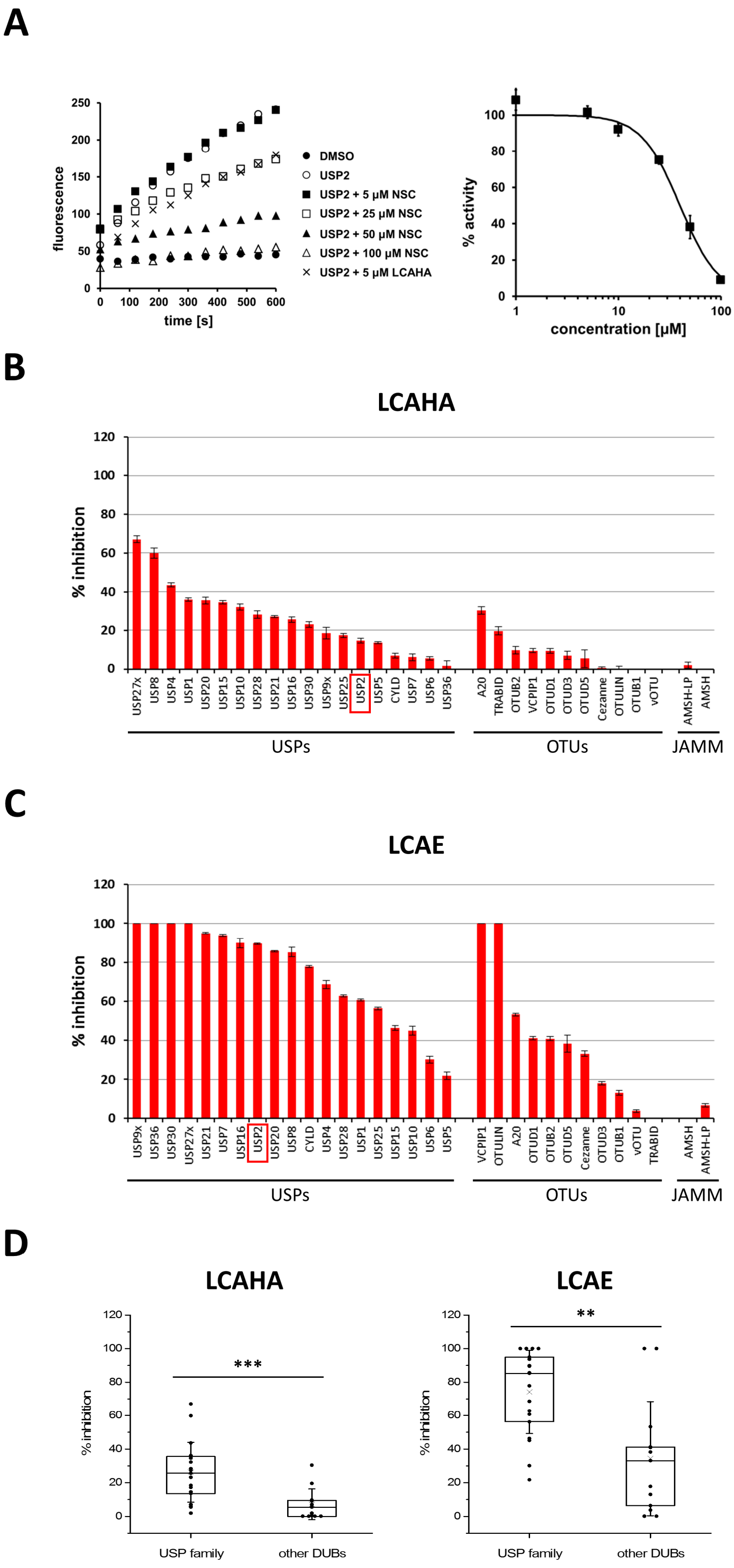
A
fluorescence
time [s]
DMSO
USP2
USP2 + 5 µM NSC
USP2 + 25 µM NSC
USP2 + 50 µM NSC
USP2 + 100 µM NSC
USP2 + 5 µM LCAHA
% activity
concentration [µM]
B
LCAHA
% inhibition
USP27x USP8 USP4 USP1 USP20 USP15 USP10 USP28 USP21 USP16 USP30 USP9x USP25 USP2 USP5 CYLD USP7 USP6 USP36
A20 TRABID OTUB2 VCPIP1 OTUD1 OTUD3 OTUD5 Cezanne OTULIN OTUB1 vOTU
AMSH-LP AMSH
USPs
OTUs
JAMM
C
LCAE
% inhibition
USP9x USP36 USP30 USP27x USP21 USP7 USP16 USP2 USP20 USP8 CYLD USP4 USP28 USP1 USP25 USP15 USP10 USP6 USP5
VCPIP1 OTULIN A20 OTUD1 OTUB2 OTUD5 Cezanne OTUD3 OTUB1 vOTU TRABID
AMSH AMSH-LP
USPs
OTUs
JAMM
D
LCAHA
LCAE
% inhibition
***
**
USP family
other DUBs

Figure S5. The analysis of the activity of NSC 632839 and the selectivity of LCAHA and LCAE compounds. Related to Figure 5.

(A) The effects of the NSC 632839 compound (inhibitor control) on the USP2a activity tested using the Ub-AMC hydrolysis assay. Left panel: The Ub-AMC hydrolysis assay demonstrates that 25 μM NSC 632839 inhibits the USP2a enzymatic activity at a similar level as 5 μM LCAHA compound. The graph shows representative data of multiple repeats. Right panel: A dose-response inhibition of USP2a activity measured in the Ub-AMC hydrolysis assay, in the presence of various concentrations of the NSC 632839 compound. The graph shows the mean ± SD values from the 3 experiments.

(B-D) High throughput MS-based enzyme activity assay was performed on a panel of DUB enzymes in a presence of 100 μM LCAHA (B) or LCAE (C) compounds. The graphs present mean ± SD values from duplicates. (D) The comparison of the inhibitory potential of the tested compounds towards USP proteins and other DUB enzymes. The box charts present 25/75th percentiles ± SD values. All data points are also presented on the chart. The statistical significance was calculated with U Mann-Whitney test: ** $p<0.01$, *** $p<0.001$.

Supplemental Tables

Kinetic constants	Lineweaver-Burk equation		
	USP2a	USP2a + 2 µM LCAHA	USP2a + 5 µM LCAHA
**K_m [µM]**	12.2 ± 2.8	12.1 ± 1.8	12.6 ± 3.0
**V_{max} [µMs^{-1}]**	$1.4 \cdot 10^{-2} \pm 2 \cdot 10^{-3}$	$9.9 \cdot 10^{-3} \pm 9.7 \cdot 10^{-4}$	$3.8 \cdot 10^{-3} \pm 1.3 \cdot 10^{-3}$

Table S1. The kinetic constants for USP2a in the presence of the LCAHA compound. Related to Figure 5.

Values are determined based on the kinetic analysis of USP2a-catalyzed Ub-AMC hydrolysis. Presented data are determined by fitting the Lineweaver-Burk equation.
