# Supplementary material for: Lithocholic Acid Hydroxyamide Destabilizes Cyclin D1 and Induces G0/G1 Arrest by Inhibiting Deubiquitinase USP2a
Source: Cell Chem Biol. 2017 Apr 20;24(4):458–470.e18. doi: 10.1016/j.chembiol.2017.03.002 (PMC5404848; doi:10.1016/j.chembiol.2017.03.002)
Supplement: Document S2. Article plus Supplemental Information [file mmc2.pdf]

Article

Cell Chemical Biology

Lithocholic Acid Hydroxyamide Destabilizes Cyclin D1 and Induces G_0/G_1 Arrest by Inhibiting Deubiquitinase USP2a

Graphical Abstract

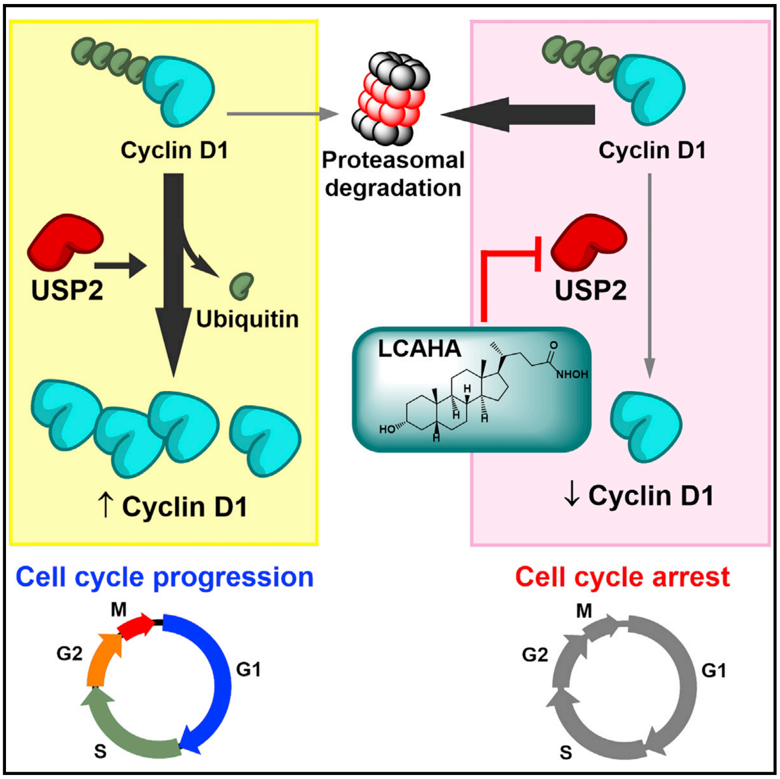

Highlights

- Lithocholic acid (LCA) derivatives are inhibitors of USP proteins in vitro
- LCA hydroxyamide (LCAHA) inhibits USP2a, leading to destabilization of cyclin D1
- LCAHA inhibits growth of cyclin D1-dependent cells at submicromolar concentrations
- Growth-inhibitory activity of LCAHA is independent of p53 status

Authors

Katarzyna Magiera, Marcin Tomala, Katarzyna Kubica, ..., Grzegorz Dubin, Tad A. Holak, Lukasz Skalniak

Correspondence

lukasz.skalniak@uj.edu.pl

In Brief

Magiera et al. demonstrate that the lithocholic acid (LCA) derivative, LCAHA, inhibits deubiquitinase USP2a. This leads to destabilization of cyclin D1 and growth inhibition of cyclin D1-dependent cells at submicromolar concentrations. The activity of LCAHA is independent of p53 status.

Magiera et al., 2017, Cell Chemical Biology *24*, 458–470
April 20, 2017 © 2017 The Authors. Published by Elsevier Ltd.
http://dx.doi.org/10.1016/j.chembiol.2017.03.002

CellPress

Please cite this article as: Magiera et al., Lithocholic Acid Hydroxyamide Destabilizes Cyclin D1 and Induces G_0/G_1 Arrest by Inhibiting Deubiquitinase USP2a, Cell Chemical Biology (2017), http://dx.doi.org/10.1016/j.chembiol.2017.03.002

Lithocholic Acid Hydroxyamide Destabilizes Cyclin D1 and Induces G_0/G_1 Arrest by Inhibiting Deubiquitinase USP2a

Katarzyna Magiera,[1,2] Marcin Tomala,[1] Katarzyna Kubica,[1] Virginia De Cesare,[3] Matthias Trost,[3] Bartosz J. Zieba,[2] Neli Kachamakova-Trojanowska,[4] Marcin Les,[1] Grzegorz Dubin,[2,5] Tad A. Holak,[1,2] and Lukasz Skalniak[1,2,6,*]

[1]Department of Organic Chemistry, Faculty of Chemistry, Jagiellonian University, ul. Ingardena 3, 30-060 Krakow, Poland
[2]Malopolska Centre of Biotechnology, Jagiellonian University, ul. Gronostajowa 7a, 30-387 Krakow, Poland
[3]MRC Protein Phosphorylation and Ubiquitylation Unit, University of Dundee, Dundee DD1 5EH, Scotland, UK
[4]Department of Medical Biotechnology, Faculty of Biochemistry, Biophysics and Biotechnology, Jagiellonian University, ul. Gronostajowa 7, 30-387 Krakow, Poland
[5]Department of Microbiology, Faculty of Biochemistry, Biophysics and Biotechnology, Jagiellonian University, ul. Gronostajowa 7, 30-387 Krakow, Poland
[6]Lead Contact
*Correspondence: lukasz.skalniak@uj.edu.pl

http://dx.doi.org/10.1016/j.chembiol.2017.03.002

SUMMARY

USP2a is a deubiquitinase responsible for stabilization of cyclin D1, a crucial regulator of cell-cycle progression and a proto-oncoprotein overexpressed in numerous cancer types. Here we report that lithocholic acid (LCA) derivatives are inhibitors of USP proteins, including USP2a. The most potent LCA derivative, LCA hydroxyamide (LCAHA), inhibits USP2a, leading to a significant Akt/GSK3β-independent destabilization of cyclin D1, but does not change the expression of p27. This leads to the defects in cell-cycle progression. As a result, LCAHA inhibits the growth of cyclin D1-expressing, but not cyclin D1-negative cells, independently of the p53 status. We show that LCA derivatives may be considered as future therapeutics for the treatment of cyclin D1-addicted p53-expressing and p53-defective cancer types.

INTRODUCTION

Lithocholic acid (LCA) is a representative of secondary bile acids. It is produced by intestinal bacteria from chenodeoxycholic acid, which is one of the final products of cholesterol catabolism (Staels and Fonseca, 2009). The primary role of bile acids is to promote the absorption of lipids, but they are also involved in the regulation of cholesterol and glucose homeostasis by the means of endocrine mechanisms (Houten et al., 2006). The biological activity of secondary bile acids in relation to carcinogenesis has been investigated previously (Nagengast et al., 1995). On one hand, an increased level of free secondary bile acids was shown to correlate with the incidence of colon cancer, and cell- and animal-based studies showed that secondary bile acids act as tumor promoters (Bernstein et al., 2011; Farhana et al., 2016; Prasad et al., 2014). On the other hand, cytotoxic properties of LCA and its diverse derivatives have been utilized in multiple approaches, aimed at the discovery of antitumor drugs. LCA became one of the new scaffolds in this area due to its biological activity, natural occurrence, and uncommon toxicity profile in that although it is toxic for small animals, it is safe for humans (Hofmann, 2004). Goldberg et al. (2011) reported that LCA induces apoptosis in neuroblastoma cell lines in a concentration range non-toxic to primary neurons, pointing to the selectivity of the compound toward cancer cells.

The toxicity of bile acids against hepatocyte- and colonocyte-derived cancer cell lines was widely studied in the past. Bile acids, including LCA, have been shown to enantioselectively induce apoptosis in human colorectal carcinoma and colorectal adenocarcinoma cell lines, yet the effective concentration of tested compounds was in a three-digit micromolar range (Katona et al., 2009). Of note, previous studies on the toxicity of deoxycholic acid and its derivatives were also performed with the use of high, submillimolar concentrations, suggesting rather weak bioactivity of tested compounds (Payne et al., 2005; Wachs et al., 2005; Yui et al., 2005). The improvement of anticancer properties of bile acid-derived compounds was only recently achieved by introducing diverse structural modifications, thus obtaining molecules active at low-micromolar concentrations (Májer et al., 2014; Sharma et al., 2010; Singh et al., 2015). Following this trend, we investigated whether more potent LCA derivatives may be obtained. We prepared a panel of LCA modifications and evaluated their biological properties in a set of in vitro tests. Our study identified growth-inhibitory properties of LCA derivative LCAHA toward HCT116 cells, unexpectedly revealing a cyclin D1 degradation-dependent mechanism of action resulting from a direct inhibition of ubiquitin-specific peptidase 2a (USP2a).

RESULTS

The Design of LCA Derivatives

In a search for new small-molecule candidates for targeting colon cancer, we have synthesized a panel of LCA derivatives, following earlier discoveries by Katona et al. (2009) and Vogel et al. (2012). We synthesized a variety of C3, C24, and double C3/C24 modifications of LCA, introducing small moieties (amine,

 © 2017 The Authors. Published by Elsevier Ltd.
This is an open access article under the CC BY license (http://creativecommons.org/licenses/by/4.0/).

Table 1. Lithocholic Acid Derivatives Tested in This Study

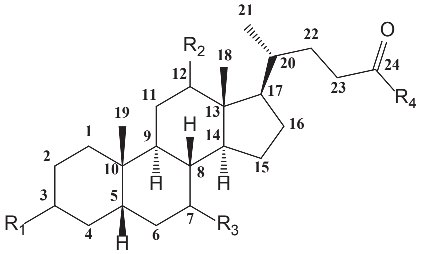

Compound	R_1	R_2	R_3	R_4
LCA	''''''''OH	–	–	—OH
CGG	''''''''OH	''''''''OH	''''''''OH	—OH
LCACN	''''''''OH	–	–	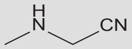
LCAE	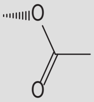	–	–	—OH
LCAGLY	''''''''OH	–	–	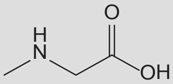
LCAK	=O	–	–	—OH
LCAME	''''''''OH	–	–	—OCH_3
LCAMR	''''''''OH	–	–	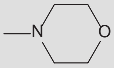
$LCANH_2$	''''''''OH	–	–	—NH_2
LCAHA	''''''''OH	–	–	—NHOH
TCAS	''''''''OH	''''''''OH	''''''''OH	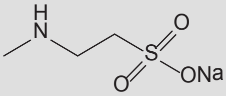
UDCA	''''''''OH	–	◀OH	—OH

carbonyl, aminonitryl) or low molecular weight esters in positions R1 and R4 (Table 1). Electronegative atoms were introduced with the aim of increasing the solubility of the compounds. In addition, by introducing the oxygen or nitrogen atoms to the steroid structure, we aimed at increasing the number of possible interactions with potential protein targets, consequently improving the biological activity of LCA. We also modified steroid carbons C3 and C24 with larger, standard solubilizing groups, such as amino ester hydrochloride, morpholine, or N-methylglucosamine. Additionally we tested the influence of hydroxyl groups at positions R2 and R3 on the biological activity by evaluating certain commercially available bile acids, namely ursodeoxycholic acid, cholic acid, and taurocholic acid.

LCA Derivatives Affect the Growth Rate and Survival of HCT116 $p53^{wt}$ and $p53^{-/-}$ Cells

To test the biological activity of the compounds, we performed MTT (3-(4,5-dimethylthiazol-2-yl)-2,5-diphenyltetrazolium bromide) and lactate dehydrogenase (LDH) release assays. In addition, the importance of p53 status was evaluated by the use of two variants of HCT116 cells: HCT116 $p53^{wt}$ (wild-type) and HCT116 $p53^{-/-}$. To track not only the toxic (lethal) but also growth-inhibitory (GI) effect in the MTT assay, we treated the cells for 6 days. In the experiment the toxic effect, but not the GI effect, was expected to be accompanied by LDH release.

All tested compounds presented lethal effect with LD_{50} values in the range 7–50 μM (for six compounds) or above 50 μM (for five compounds) (Table 2 and Figure S1). LD_{50} values were not dependent on the status of p53.

For one LCA derivative, LCAHA, a biphasic response was observed for both cell types tested: a low-concentration GI effect and a high-concentration toxic effect (Figure 1A). LDH release was observed only at the concentrations corresponding to the high-concentration toxic effect (Figure 1B). The GI_{50} values calculated from MTT results were below 1 μM for both cell lines (Table 2) and the growth of cells was inhibited by about 60% in

Table 2. Effects of Lithocholic Acid and Its Derivatives on Cell Viability and on the Enzymatic Activity of USP2a

	Cell Lines Data: MTT Assay				USP2a Activity: Enzyme Assay	
	HCT116^{wt}		HCT116 p53$^{-/-}$		Ub-AMC Assay	Di-Ub Assay
Compound	LD_{50} (μM)	GI_{50} (μM)	LD_{50} (μM)	GI_{50} (μM)	IC_{50} (μM)	IC_{50} (μM)
LCA	46.8 ± 2.6	–	37.4 ± 3.8	–	31.1 ± 3.4	32.0 ± 3.2
CGG	>50	–	>100	–	not active	>100
LCACN[a]	28.0 ± 0.7	–	29.9 ± 3.2	–	7.4 ± 1.2	13.9 ± 2.3
LCAE	23.2 ± 0.0	–	24.4 ± 0.4	–	5.8 ± 0.7	3.3 ± 0.1
LCAGLY	>100	–	>100	–	>50	>50
LCAK	>50	–	>50	–	32.0 ± 1.8	33.1 ± 3.2
LCAME[a]	20.5 ± 1.1	–	35.1 ± 4.8	–	>50	>50
LCAMR[a]	8.2 ± 0.7	–	7.9 ± 0.7	–	>100	>100
$LCANH_2$	24.5 ± 0.9	–	41.8 ± 2.7	–	27.9 ± 1.0	16.7 ± 1.3
LCAHA	27.8 ± 3.9	0.87 ± 0.09	26.5 ± 0.1	0.96 ± 0.29	9.7 ± 1.5	3.7 ± 0.8
TCAS	>50	–	>100	–	not active	>100
UDCA	>50	–	>100	–	not active	>100
NSC 632839	–	–	–	–	39.1 ± 6.4	>50

[a]Compounds for which solubility problems were encountered in either the cell line experiment or the enzymatic assay.

the concentration range 2–20 μM (Figure 1A). For the lethal effect, LD_{50} values were above 25 μM for both cell lines, giving selectivity of the GI effect over the lethal effect of 31.9-fold for p53^{wt} and 27.6-fold for p53$^{-/-}$ cells (Table 2).

To evaluate whether the observed effects are accompanied by the induction of apoptosis, we examined the activation of caspases 3 and 7 in HCT116 p53^{wt} cells. A weak and non-significant increase of caspase activity was detected in the cells treated with 1 or 5 μM LCAHA for 48 or 72 hr. Only at 10 μM LCAHA was the increase of caspase activity significant (Figure 1C). Thus, apoptosis is not efficiently induced in the GI concentration range of the compound. Treatment with staurosporine, a known inducer of apoptosis, led to a strong increase of the activity of caspases 3 and 7 (Figure 1C).

LCAHA Arrests HCT116 Cells in G_0/G_1

Low concentrations of LCAHA inhibit the growth of HCT116 cells without affecting their survival. To investigate this effect, we analyzed the cell-cycle distribution. Cells treated for 48 hr with LCAHA or DMSO were pulse-labeled with BrdU for 1 hr and stained with fluorescein isothiocyanate (FITC)-anti-bromodeoxyuridine (BrdU) antibody and propidium iodide (PI). Flow cytometry analysis revealed mild, but significant G_0/G_1 arrest of both HCT116 p53^{wt} and HCT116 p53$^{-/-}$cells treated with 5 or 10 μM LCAHA (Figure 1D). The analysis of BrdU incorporation revealed a dose-dependent decrease in the rate of DNA synthesis in LCAHA-treated cells (Figure 1E). This suggests that besides G_0/G_1 arrest, LCAHA also affects the progression through the S phase.

To verify this, we pulse-labeled HCT116 p53^{wt} cells treated with LCAHA for 48 hr for 1 hr with BrdU and harvested or cultured them for an additional 3, 6, or 9 hr. To track the progression through the S phase and G_0/G_1 arrest precisely, we analyzed FITC-positive and -negative cells separately for cell-cycle distribution using ModFit LT software. FITC-positive cells represented the cells that incorporated BrdU (the population in the S phase during pulse-labeling that progressed toward G_2/M in succeeding hours), and FITC-negative cells represented the cells that did not incorporate BrdU (the population that was either in G_0/G_1 or G_2/M phase during pulse-labeling and entered S phase or divided in succeeding hours).

As expected, the majority of BrdU$^+$ cells were in the S phase at time point t_0, and the majority of BrdU$^-$ cells were in G_0/G_1 or G_2/M phases (Figure 1F). Nine hours later only 23% of BrdU$^+$ cells treated with DMSO were still in the S phase and more than 50% of cells had finished the cell cycle and were located in G_0/G_1 phase (Figure 1F, top panel). The treatment with LCAHA dose-dependently inhibited the transition of BrdU$^+$ cells through the S phase, as evidenced by the significantly higher proportion of cells remaining in the S phase (up to 56% at 5 μM LCAHA) and significantly lower proportion of cells in G_0/G_1 phase (down to 24%). At the same time, around 44% of BrdU$^-$ cells treated with DMSO left G_0/G_1 phase and entered the S phase, while treatment with LCAHA significantly and dose-dependently inhibited this transition down to 20% at 5 μM concentration of the compound (Figure 1F, bottom three panels). These results reveal a much stronger G_0/G_1 arrest than suggested by a simple analysis of the whole population of the cells (Figure 1C). The complete outcome of the test, presenting the progress of the cell cycle with 3-h intervals, is presented in Figure S2.

LCAHA-Induced G_0/G_1 Arrest Is Accompanied by Decreased Expression of Cyclin D1

The progression from G_1 to S phase relies largely on the expression and activity of D cyclins. To verify whether the observed impairment in cell-cycle progression following LCAHA treatment is associated with D cyclins, we investigated the expression of cyclins D1 and D3 in HCT116 p53^{wt} and HCT116 p53$^{-/-}$ cells. A significant, dose-dependent decrease of cyclin D1 expression was observed in both cell lines treated with 5 or 20 μM LCAHA; however, this effect was more evident in HCT116 p53^{wt} cells (Figure 2A). LCA did not affect the expression of cyclin D1, and

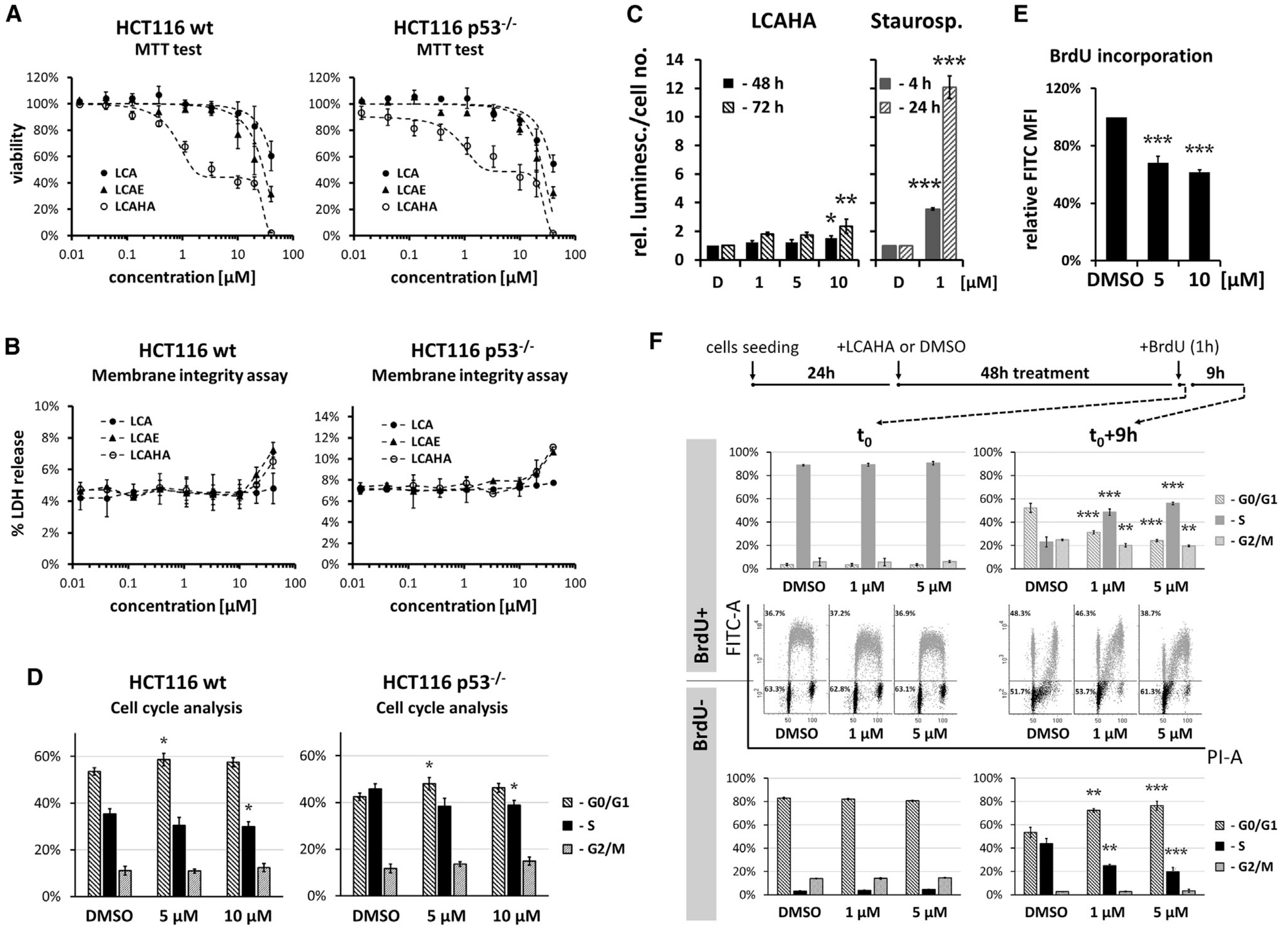

Figure 1. LCAHA Inhibits the Growth of HCT116 Cells

(A) MTT assay. The cells were seeded at low confluence and treated with indicated compounds for 6 days. Cell viability was assayed by MTT. The p53 status of tested cells is indicated. See also Figure S1.

(B) LDH release assay was performed following 48 hr of treatment. The data are presented as a percentage of total LDH activity in the treated cells lysed with Triton X-100.

(C) The activity of caspases 3 and 7 was measured in HCT116 $p53^{wt}$ cells treated with LCAHA or staurosporine for the indicated time periods. The graphs present the results normalized to cell numbers at the time of caspase activity testing and then to DMSO-treated controls.

(D–F) The analysis of cell cycle was performed on HCT116 $p53^{wt}$ (D [left panel], E, and F) and HCT116 $p53^{-/-}$ (D, right panel) cells treated with DMSO or LCAHA for 48 hr and pulse-labeled with BrdU. The cells were harvested or cultured for additional 9 hr in the absence of BrdU. The cells were stained with FITC-conjugated anti-BrdU antibody and propidium iodide (PI), and analyzed by flow cytometry for cell-cycle distribution (D and F) or BrdU incorporation (E). (F) The analysis of cell-cycle distribution was performed separately for $BrdU^+$ (blue) and $BrdU^-$ (black) populations of cells. For the detailed description of the experiment, see also Figure S2.

All graphs show mean values ± SD from three independent experiments. Statistical significance was evaluated using one-way ANOVA with Tukey's post hoc test: $^*p < 0.05$, $^{**}p < 0.01$, $^{***}p < 0.001$.

LCAE, which did not present a biphasic effect on the survival of HCT116 cells, decreased the expression of the protein in HCT116 $p53^{wt}$ cells, but this effect was much weaker than for LCAHA (Figure 2A). A 24-hr treatment with any of the compounds did not affect the expression of cyclin D1 in HCT116 cell lines (Figure S3B).

The expression of cyclin D3 was significantly increased in both HCT116 $p53^{wt}$ and HCT116 $p53^{-/-}$ cells treated with LCA, but the treatment with LCAE or LCAHA had no effect on the expression of cyclin D3 (Figures 2A and S3A). Also, the expression of p27, a negative regulator of cyclins, was not altered by LCA, LCAE, or LCAHA (Figures 2A and S3A).

The treatment of HCT116 $p53^{wt}$ cells with LCA, LCAE, or LCAHA for 24 or 48 hr did not induce the expression of proteins encoded by p53-target genes (MDM2 and p21) or alter the expression of the p53 protein itself (Figure S3B). This suggests that up to 20 µM concentration, the effect of the tested compounds on the HCT116 growth is independent of p53.

Cyclin D1 Is Crucial for the Activity of LCAHA

To verify the importance of cyclin D1 for the activity of LCAHA, we tested an additional three cell lines: breast adenocarcinoma cell line MCF-7 (cyclin D1 dependent [Grillo et al., 2006]), osteosarcoma cell line U-2 OS (cyclin D1 positive), and osteosarcoma

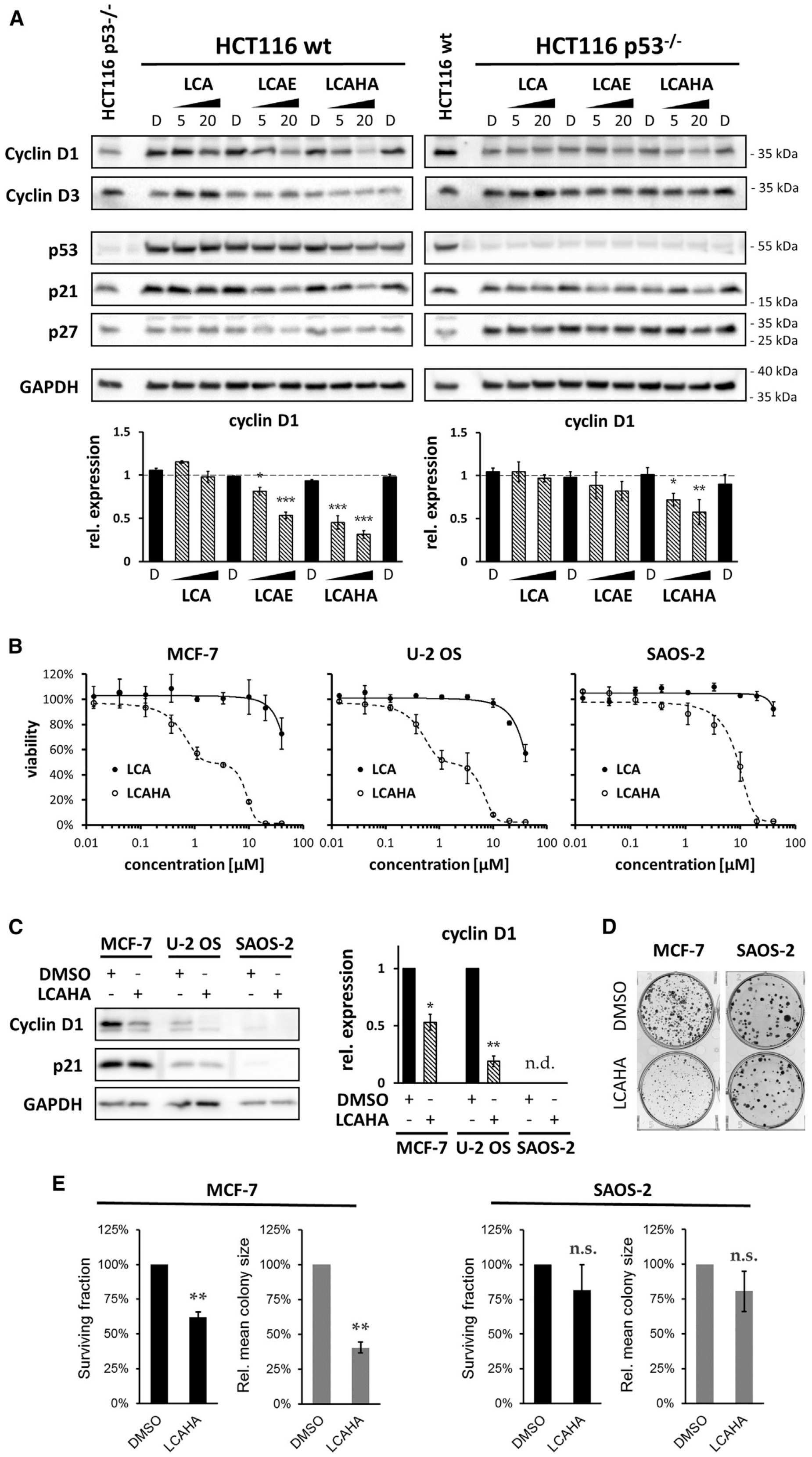

(legend on next page)

cell line SAOS-2 (cyclin D1 negative). For MCF-7 and U-2 OS cells, treatment with LCAHA resulted in a biphasic, dose-dependent decrease of viability, similar to the result observed for HCT116 cells (Figure 2B). In the case of SAOS-2 cells, the viability decrease was observed only at high concentrations of compound (Figure 2B). LCA was much less active for all three cell lines (LD_{50} > 50 μM). For both MCF-7 and U-2 OS cell lines a significant decrease of cyclin D1 expression was observed following LCAHA treatment, while the expression of cyclin D1 was not observed in SAOS-2 cells (Figure 2C). In addition a colony-forming assay was performed on cyclin D1-addicted MCF-7 cells and cyclin D1-negative SAOS-2 cells. LCAHA treatment did not affect the clonogenic potential of SAOS-2 cells, while for the MCF-7 cells a significant decrease of the clonogenic potential was observed, as revealed by analysis of the surviving fraction and mean colony size (Figures 2D and 2E).

To additionally assess the importance of cyclin D1 for the activity of LCAHA, we knocked down the expression of cyclin D1 in the HCT116 $p53^{wt}$ using small interfering RNA (siRNA) technology, and treated the cells with LCAHA. Analysis of cell-cycle progression was performed following double staining with the PI- and FITC-conjugated anti-BrdU antibody, as described above. The knockdown of cyclin D1 was verified with western blotting (Figure 3, left panel).

In the absence of cyclin D1 the growth of HCT116 cells was reduced by around 30% (not shown). However, at the time of the analysis (72 hr post transfection) the progression through the cell cycle was similar for both the knocked down cyclin D1 and control cells (Figure 3, DMSO-treated cells: samples 1–4). Apparently, the cells adapt to cyclin D1 deprivation as described previously (Sherr and Roberts, 2004). In fact, cyclin D1 knockdown was assisted by the decreased expression of p27, which may improve the cycling of cyclin D1-deprived cells (Figure 3, left panel).

Treatment with LCAHA blocked the cell-cycle progression of the control siRNA-transfected HCT116 cells (Figure 3, compare sample 6 with sample 2). Importantly, the knockdown of cyclin D1 partially restored normal cycling of the cells treated with LCAHA. In the absence of cyclin D1 significantly more of the LCAHA-treated $BrdU^-$ cells entered the S phase, and significantly more of the LCAHA-treated $BrdU^+$ cells finished the cell cycle, compared with the cells with normal cyclin D1 expression (Figure 3, compare samples 8 and 6).

LCAHA Antagonizes the Mitogenic Environment

Cyclin D1 is a key modulator of the transition through the G_1/S checkpoint and is known to be sensitive to proliferative signals delivered by the mitogens. To evaluate the interplay between the mitogens provided by the serum and LCAHA, we performed a synchronization-release assay on the cells treated with LCAHA. To assess the involvement of cyclin D1 in this interplay, we used MCF-7 (cyclin D1-dependent) and SAOS-2 (cyclin D1-negative) cells. The cells were synchronized in G_0/G_1 by the removal of fetal bovine serum (FBS) for 2 days of the culture in the presence of LCAHA. After this time, the cells were released by the addition of medium containing 10% FBS for 24 hr (still in the presence of the compound).

Serum starvation resulted in an increase of the proportion of cells in G_0/G_1 phase at the expense of the S phase, while the addition of serum resulted in a gross entry into the S phase for both cell lines treated with DMSO, as expected (Figure S4). Pre-treatment with LCAHA significantly inhibited the entry into the S phase in MCF-7 but not in SAOS-2 cells (Figure S4). This result confirms that LCAHA blocks the proliferative signals delivered by serum and suggests the involvement of cyclin D1 in this phenomenon.

LCAHA Lowers Cyclin D1 Half-Life Despite Increased Phosphorylation of Akt and GSK-3β

The presence of serum provides proliferative signals by activating ERK and AKT signaling pathways, which increase the expression of cyclin D1 (Tari and Lopez-Berestein, 2000). ERK activation induces de novo synthesis of cyclin D1 (Chambard et al., 2007), while AKT is involved in cyclin D1 stabilization by alleviating a destabilizing impact of GSK-3β kinase (Diehl et al., 1998).

To characterize the mechanism of cyclin D1 downregulation by LCAHA, we first examined the level of *CCND1* mRNA. In the three cell lines, i.e., HCT116, MCF-7, and U-2 OS, *CCND1* mRNA was detected, but its level did not change after the treatment with LCAHA (Figure 4A). In SAOS-2 cells *CCND1* mRNA was not detected.

We then verified the stability of cyclin D1 in LCAHA-treated HCT116 cells. The cells were treated for 48 hr with DMSO or 5 μM LCAHA, and cycloheximide (CHX) was applied for the last 15–60 min of the treatment. The half-life of the protein was significantly decreased ($p = 0.025$) from 40.6 ± 2.4 min in the DMSO-treated cells to 25.3 ± 2.0 min in LCAHA-treated cells (Figures 4B and 4C).

To assess the involvement of AKT pathway in the observed decrease of cyclin D1 stability, we monitored the phosphorylation of Akt kinase and its direct target GSK-3β along with the dynamics of cyclin D1 decay in HCT116 $p53^{wt}$ cells. The cells were

Figure 2. The Involvement of Cyclin D1 in the Growth-Inhibitory Effect of LCAHA

(A) HCT116 $p53^{wt}$ (left panel) or HCT116 $p53^{-/-}$ (right panel) cells were treated with DMSO (marked with D), or 5 or 20 μM tested compounds for 48 hr, followed by western blot analysis. The graphs present densitometry analysis of cyclin D1 expression and show mean ± SEM from three independent experiments. Statistical significance was evaluated using one-way ANOVA with Tukey's post hoc test: *$p < 0.05$, **$p < 0.01$, ***$p < 0.001$. See also Figure S3.

(B) For the MTT assay the cells were seeded at low confluence and treated with tested compounds for 6 days. The graphs show mean values ± SD from three independent experiments.

(C) Analysis of cyclin D1 and p21 expression in the cells treated with DMSO or 10 μM LCAHA for 48 hr. The graph presents densitometry analysis of cyclin D1 expression and shows mean ± SEM from three independent experiments. For the statistics, a t test was performed: *$p < 0.05$, **$p < 0.01$.

(D) Colony formation assay was performed on MCF-7 or SAOS-2 cells treated with DMSO or 5 μM LCAHA for 5 days. The photographs are representative of three experiments.

(E) The numbers and sizes of the colonies formed in the colony formation assay (D) were measured using ImageJ software. Based on these data, the surviving fraction and relative mean colony size was calculated for the LCAHA-treated cells. The graphs show mean ± SE values from three experiments. For the statistics, a t test was performed: **$p < 0.01$; n.s., not significant.

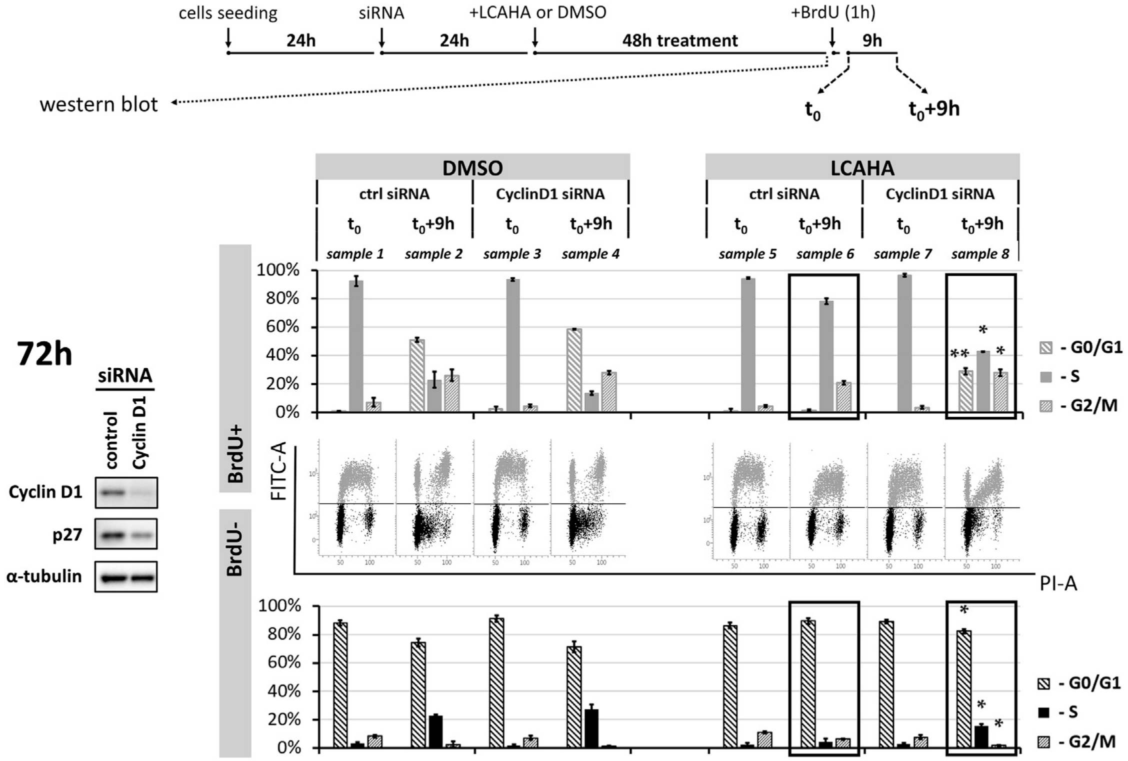

Figure 3. Cyclin D1 Knockdown Renders HCT116 Cells Resistant to LCAHA

HCT116 p53[wt] cells were transfected with the cyclin D1 siRNA or control siRNA, followed by treatment with 5 μM LCAHA or DMSO as a control, as presented on the scheme at the top of the figure. Silencing of the cyclin D1 expression was verified 48 hr following treatment (72 hr following transfection) by western blotting (left panel). At the same time, the cells were pulse-labeled with BrdU and immediately harvested (time point "t_0") or cultured for an additional 9 hr (time point "t_0+9h"). The cells were stained with the PI- and with FITC-conjugated anti-BrdU antibody and analyzed by flow cytometry. $BrdU^+$ and $BrdU^-$ cells were analyzed separately for cell-cycle distribution using ModFit LT software. The dot-plot graphs show the representative results of two experiments, and the bar graphs show mean ± SD from these experiments. The samples indicated with the black rectangles were analyzed for statistically significant differences using the t test: *$p < 0.05$, **$p < 0.01$. See also Figure S4.

treated for 24, 26, 28, 30, or 32 hr with LCAHA or DMSO. A significant decrease in cyclin D1 was observed over the time course of the experiment (Figures 4D and 4E). Surprisingly, this was accompanied by an increase of the phosphorylation of both Akt and GSK-3β, which unexpectedly suggests a positive impact of the AKT pathway on cyclin D1 protein stability (Figures 4D and 4E).

LCA and Its Derivatives Inhibit the Activity of USP2a

In 2009 Shan and colleagues demonstrated that USP2a deubiquitinase stabilizes cyclin D1 by removing ubiquitin moieties, thus protecting the protein from proteasomal degradation (Shan et al., 2009). To verify the engagement of USP2a in the action of LCAHA, we first looked at the expression of two other known targets of USP2a deubiquitinase: Aurora A (Shi et al., 2011) and cyclin A1 (Kim et al., 2012). HCT116 cells were treated for 48 hr with DMSO or LCAHA at two concentrations, 5 μM and 20 μM. A significant decrease of the expression level was observed for both evaluated proteins following LCAHA treatment (Figure 4F). This observation supports the notion that LCAHA inhibits USP2a in HCT116 cells.

To verify the cell line data, we tested in vitro the ability of LCA and its derivatives to directly inhibit USP2a activity in Ub-AMC hydrolysis and FRET (fluorescence resonance energy transfer) Di-Ub K63-2 assays. An active, histidine-tagged USP2a catalytic domain was pre-incubated with various concentrations of LCA and its derivatives for 30 min and the rate of hydrolysis of substrates was measured. Both assays yielded comparable results. LCA and its five derivatives inhibited USP2a with IC_{50} values in the range 2–37 μM (Table 2). The most potent compounds, LCAE and LCAHA, exhibited IC_{50} values in a one-digit micromolar range (Table 2 and Figures 5A–5C). The IC_{50} values determined for LCACN are similar to those for LCAE and LCAHA, although because of the observed solubility problems these values are not reliable. The remaining compounds showed low or no activity.

For comparison, previously described deubiquitinase (DUB) inhibitor NSC 632839 was tested in Ub-AMC and Di-Ub K63-2 assays yielding IC_{50} values of 39.1 ± 6.4 μM and above 50 μM, respectively (Figure S5A). This was in agreement with the previously reported EC_{50} value of 45 ± 4 μM (Nicholson et al., 2008).

To additionally verify the binding of LCAE and LCAHA to USP2a, we performed a fluorescence-based thermal shift assay (Pantoliano et al., 2001). In this assay the interaction with small-molecule ligands induces a thermal stabilization of the protein,

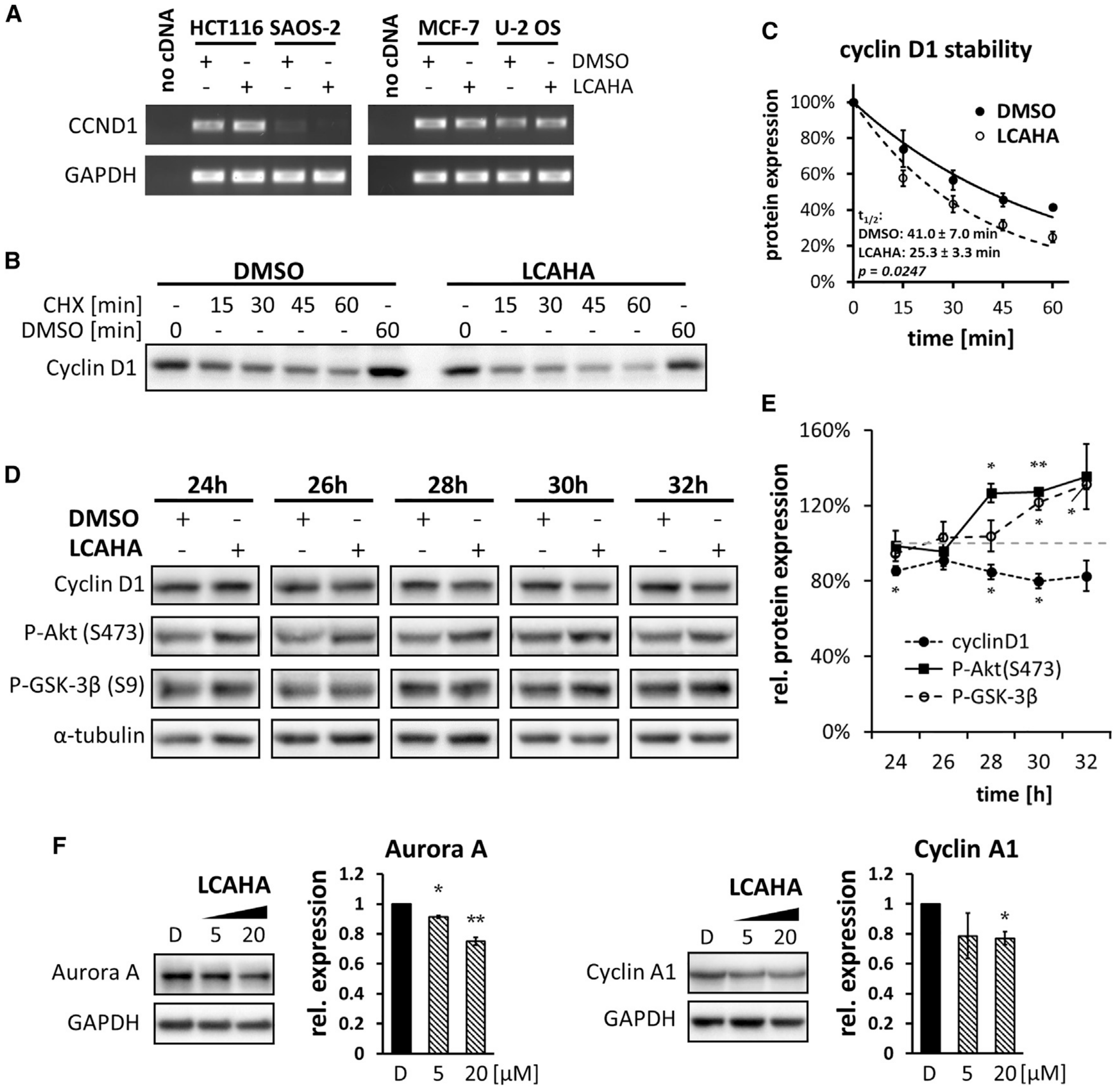

Figure 4. Impact of LCAHA on the Expression and Stability of Cyclin D1

(A) The expression of cyclin D1-encoding mRNA (*CCND1*) was evaluated by RT-PCR. The cells were treated with DMSO or 10 μM LCAHA for 48 hr.

(B and C) The cells were treated with DMSO or LCAHA for 48 hr, and cycloheximide (CHX) was added for the last 15–60 min, followed by western blot analysis of cyclin D1 expression. The graph in (C) presents densitometry analysis of the expression level of cyclin D1 (B) and shows mean ± SEM values from three independent experiments. The statistical significance was evaluated using a t test on mean $t_{1/2}$ values from these three experiments.

(D and E) Western blot analysis of the expression of cyclin D1, phospho-Akt, and phospho-GSK-3β in HCT116 $p53^{wt}$ cells after the treatment with 5 μM LCAHA for the indicated time periods. (D) Western blot images from the representative experiment. (E) Densitometry analysis of the expression level of cyclin D1, P-Akt(S473), and P-GSK-3β(S9) normalized to α-tubulin. The graphs show mean ± SD from three independent experiments. Statistical significance was evaluated using a t test: $^*p < 0.05$, $^{**}p < 0.01$.

(F) HCT116 cells were treated with DMSO (marked with D), or 5 or 20 μM LCAHA for 48 hr, followed by western blot analysis of the expression of Aurora A and cyclin A1. The graphs present densitometry analysis of the proteins' expression and show mean ± SEM from three independent experiments. Statistical significance was evaluated using one-way ANOVA with Tukey's post hoc test: $^*p < 0.05$, $^{**}p < 0.01$.

which is observed as a change in the protein melting point proportional to the affinity of the molecule (Matulis et al., 2005).

The measured melting temperature of USP2a protein was relatively low, with a T_m value of 35.4°C (Figure 5D). LCAE and LCAHA increased the melting point of USP2a by 4.6°C (T_m 40.0°C) and 1.8°C (T_m 37.2°C), respectively (Figure 5D). The data confirm the interaction of tested compounds with the protein. Furthermore, the results show that LCAE exhibits better affinity to USP2a compared with LCAHA, in agreement with our in vitro enzyme activity assays.

To characterize the mode of action of LCAHA, we studied the kinetics of USP2a inhibition by performing Ub-AMC assay at varied substrate and inhibitor concentrations. The kinetics constants were determined using a linear regression curve fitting to the double reciprocal Lineweaver-Burk plot (Figure 5E). The determined V_{max} values decreased significantly with the

Please cite this article as: Magiera et al., Lithocholic Acid Hydroxyamide Destabilizes Cyclin D1 and Induces G_0/G_1 Arrest by Inhibiting Deubiquitinase USP2a, Cell Chemical Biology (2017), http://dx.doi.org/10.1016/j.chembiol.2017.03.002

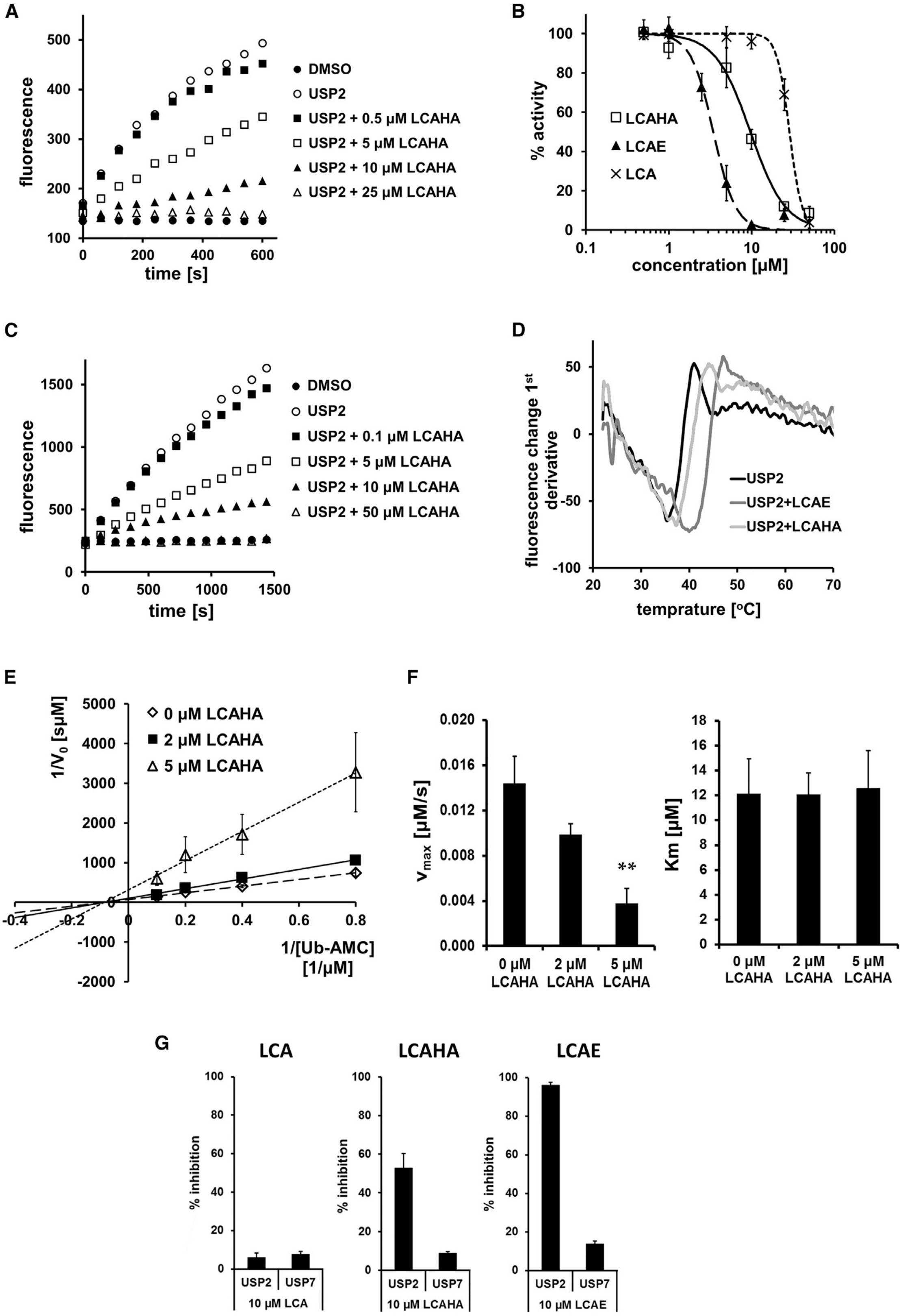

(legend on next page)

increasing concentration of LCAHA, while K_m values remained unchanged (Figure 5F and Table S1), suggesting a non-competitive mode of inhibition.

LCAE and LCAHA Are Selective Inhibitors of USP Proteins

To test the selectivity of LCA and its most potent derivatives LCAE and LCAHA, we tested the inhibition of USP7 protein activity in a Ub-AMC assay. LCA showed no inhibition, while LCAHA and LCAE demonstrated a selective inhibition of USP2a protein at the concentration of 10 μM (Figure 5G).

In addition, we tested the activity of LCAE and LCAHA toward a panel of DUBs using a mass spectrometry-based high-throughput enzyme activity assay (Ritorto et al., 2014). In the experiment 32 DUB enzymes belonging to three separate families were used: ubiquitin-specific proteases (USPs, 19 proteins), ovarian tumor proteases (OTUs, 11 proteins), and JAMM/MPN$^+$ (2 proteins).

For both compounds the inhibition of a subset of DUB enzymes was observed. Similarly to our previous experiments, LCAHA presented lower activity than LCAE (Figures S5B and S5C). The analysis of the impact of the compounds on three separate DUB families, USPs, OTUs, and JAMM/MPN$^+$ proteins, revealed a significant selectivity of LCAHA and LCAE toward USP proteins (Figure S5D, $p = 0.0006$ for LCAHA and $p = 0.0022$ for LCAE). Besides the two exceptions for both LCAHA and LCAE (i.e., A20 and TRABID for LCAHA, and VCPIP1 and OTULIN for LCAE), the activity of OTU enzymes was much less affected by the compounds than the activity of USP enzymes. The activity of neither AMSH nor AMSH-LP, both belonging to JAMM/MPN$^+$ family, was inhibited by the two tested compounds. This indicates that the compounds show selectivity toward USP enzymes, and confirm that USP2 is among the enzymes most potently inhibited by the compounds.

DISCUSSION

Bile acids are considered promising scaffolds for the design of antitumor compounds. In the present study, we describe the synthesis and characteristics of several LCA derivatives, including LCAHA, which demonstrated a unique ability to selectively inhibit the growth of cyclin D1-expressing cell lines. The GI activity of LCAHA was not associated with the induction of necrosis or apoptosis, as demonstrated by the analysis of the activity of caspases 3 and 7 and LDH release. Instead, an inhibition of cell growth was observed at a non-toxic concentration range of 0.5–15 μM in cyclin D1-expressing, but not cyclin D1-negative cells.

Cyclin D1 was reported to function as an oncoprotein, providing the cancer cell not only with the advantage of mitogen-independent proliferation but also modulation of processes such as angiogenesis and DNA-damage response (Bartkova et al., 1995; Musgrove et al., 2011). In normal cells cyclin D1 is a short-lived protein with a half-life of around 30 min (Diehl et al., 1997). Its stability is tightly regulated by multiple cellular kinases, including protein kinase GSK3β, which phosphorylates cyclin D1, targeting the protein to proteasomal degradation (Alao, 2007; Diehl et al., 1998). The activity of GSK3β is inhibited by the Akt kinase-driven phosphorylation, activated in response to mitogenic stimuli. The treatment of HCT116 cells with LCAHA significantly decreased the half-life of cyclin D1, suggesting that LCAHA affects the mechanism of cyclin D1 degradation. Interestingly, instead of AKT pathway inhibition we observed an increased phosphorylation of both Akt and GSK3β kinases, suggesting a cyclin D1-stabilizing rather than -destabilizing environment (Shimura et al., 2012).

In 2009 a novel component regulating cyclin D1 expression was described by Shan et al., who identified USP2a deubiquitinase as an enzyme responsible for cyclin D1 stabilization. Our biochemical analysis demonstrated that LCA and its derivatives inhibit USP2a activity. The decreased expression level of two other USP2a targets, namely Aurora A and cyclin A1, confirmed the inhibition of USP2a by LCAHA in the cells. LCA itself was a relatively weak inhibitor of USP2a in the in vitro DUB activity assay. Replacing the hydroxyl groups at positions R_1 and R_4 with methyl acetate or amine moieties (LCAE and LCAHA, respectively) strongly improved the potency of the tested compounds in USP2a inhibition. LCAE demonstrated the best USP2a inhibition in biochemical assay, but no GI properties in cell-based assay. We hypothesize that this was due to the partial hydrolysis of an ester bond in the cellular environment, leading to the release of LCA, which was inactive in the inhibition of cell growth. The introduction of larger substituents at position R_4, and additional hydroxyl

Figure 5. Effect of Selected Compounds on USP2a Activity

(A) Ub-AMC hydrolysis assay demonstrates that LCAHA inhibits USP2a enzymatic activity. USP2a catalytic domain was incubated with the substrate and increasing concentrations of LCAHA and the fluorescence signal were measured during the time course of the experiment. The graph shows representative data of three or more repeats. See also Figure S5.

(B) Dose-response inhibition of USP2a activity measured in the Ub-AMC hydrolysis assay in the presence of various concentrations of LCA and its derivatives LCAE and LCAHA. The graph shows mean ± SD values from three independent experiments. See also Figure S5.

(C) Di-Ub K63-2 hydrolysis assay demonstrates that LCAHA inhibits USP2a capability to hydrolyze the isopeptide bond between two ubiquitin molecules. The graph shows representative data of three or more repeats.

(D) LCA derivatives induce thermal stabilization of USP2a, as monitored by thermal shift assay. The graph presents the effect on USP2a thermal stabilization in the presence of 50 μM LCAE or LCAHA.

(E) Kinetic analysis of the USP2a-catalyzed hydrolysis of Ub-AMC substrate in the presence of the LCAHA. The reciprocal initial velocities are plotted versus the reciprocal substrate concentrations (Lineweaver-Burk plot). The graphs show mean ± SD values from three independent experiments.

(F) V_{max} and K_m values determined by fitting the Lineweaver-Burk equation. Kinetic constants trends are characteristic for the non-competitive inhibition model. The graphs show mean ± SD values from three independent experiments. Statistical significance was evaluated using one-way ANOVA with Tukey's post hoc test: $^{**}p < 0.01$. See also Table S1.

(G) The inhibition of USP2a and USP7 enzymes by the LCA, LCAE, and LCAHA compounds in the Ub-AMC assay. The graphs present percent inhibition of the proteins' activity in the presence of compounds at 10 μM and show mean ± SD values from three independent experiments. See also Figure S5.

groups in positions R_2 and R_3, resulted in low or no activity of the compounds.

To date, three distinct classes of small-molecule inhibitors of USP2a have been reported: NSC 632839 (Nicholson et al., 2008), chalcones (Issaenko and Amerik, 2012), and the compound ML364 (Davis et al., 2016), with activities at one- or two-digit micromolar concentrations. Of note, the first two classes of the inhibitors are covalent, cysteine-reactive electrophiles, while the latter is a non-covalent inhibitor but with the exact mechanism of action unknown. For LCAHA, the analysis of the kinetics of Ub-AMC hydrolysis by USP2a revealed a decrease in V_{max} and no change in K_m values. These trends are characteristic for a non-competitive inhibition model, which assumes that the inhibitor interacts with the enzyme at a location other than the active site, resulting in the slowing down of the reaction rate.

Cyclin D1 acts in concert with p27 in regulating cell-cycle progression in dividing cells (Sa et al., 2005). In our study, the treatment with LCAHA markedly decreased the expression of cyclin D1 while the expression of p27 was unchanged. This causes an increased p27/cyclin D1 ratio, which has been reported to prolong G_1 phase and promote G_0/G_1 cell-cycle arrest (Sa et al., 2005; Stacey, 2010). Accordingly, we observed G_0/G_1 arrest in HCT116 cells treated with the compound.

Along with G_0/G_1 arrest, a slower progression through the S phase was observed in LCAHA-treated cells, which obviously masked the real extent of G_0/G_1 arrest. It seems that the combination of G_0/G_1 arrest and slower progression through the S phase together contribute to the prolongation of the cell cycle, resulting in cell-growth inhibition observed in a non-toxic concentrations of LCAHA.

While the G_0/G_1 arrest is a clear consequence of a decreased cyclin D1/p27 expression ratio, the inhibition of the rate of DNA synthesis may be confusing (Fukami-Kobayashi and Mitsui, 1999). However, decreased cyclin D1 in G_1 phase may have two consequences, which may affect the time required for DNA duplication. First, cyclin D1 is required for the expression of cyclin E, which participates in the initiation of DNA synthesis (Coverley et al., 2002; Jackson et al., 1995). Indeed, our results show that low cyclin D1 expression is accompanied by low cyclin E1. Second, cyclin D1 was shown to sequester inhibitory p27 protein from the complexes of cyclin E1 with Cdk2. Therefore, the decreased cyclin D1 level may provide more p27 for the inhibition of cyclin E1/Cdk2 complexes, thus additionally disabling the activity of the already depleted cyclin E1 (Cheng et al., 1998). Although the study of the cyclin D1 knockdown presented herein suggests at least partial engagement of cyclin D1 in both described effects, some further hints on the mechanism of the inhibition of DNA synthesis could include LCAHA impact on other deubiquitinases or the degradation of other USP2a-targets, which is currently the focus of our studies.

The analysis of the activity of LCAHA and LCAE toward 32 DUB enzymes from three families revealed significant specificity of the compounds toward USPs, including USP2a. We are currently exploring the structural features of USPs, which determine the observed specificity. Based on our kinetic studies the compounds are not expected to bind at the active site of USP2a, but since only the catalytic domain of USP2a was used in our biochemical assays, the compounds bind to this domain of the USPs. Therefore, our study of the specificity of LCA derivatives toward DUB enzymes may ultimately lead to specific non-competitive inhibitors in the future.

In previous studies, the toxic and pro-apoptotic properties of LCA derivatives have been reported to be beneficial for the elimination of cancer cells (Goldberg et al., 2011; Katona et al., 2009; Májer et al., 2014; Sharma et al., 2010; Singh et al., 2015). These studies, however, did not decipher the molecular targets with a single exception whereby LCA was shown to activate p53 through antagonizing MDM2 and MDMX (Vogel et al., 2012). Notably, in our experiments performed at relatively low concentrations of LCA and its derivatives, no effect on p53 activity was found. This was shown by testing the expression of proteins encoded by p53-regulated genes p21 and MDM2, as well as the expression of p53 itself. The tested compounds demonstrated similar dose-response curves regardless of p53 status in the HCT116 cell line. Therefore, it seems that at concentrations up to 20 μM the effect of the tested compounds on cell growth is p53 independent.

In summary, we show that cell-growth inhibition induced by LCAHA is accompanied by a significant decrease in cyclin D1 level, which is a consequence of decreased stability of the protein. We also show that LCAHA antagonizes proliferative signals delivered by the presence of serum in the culture medium and describe the mechanism of engagement of USP2a protein in the observed destabilization of cyclin D1 by LCAHA. Our work constitutes a starting point for the design of more potent USP inhibitors based on an LCA scaffold for improved treatment of cancer.

SIGNIFICANCE

Cytotoxic properties of lithocholic acid (LCA) have been utilized in multiple approaches aimed at the discovery of antitumor drugs. Structure optimization of LCA led to the discovery of molecules with improved anticancer properties, active at low-micromolar concentrations. In the present study, we report for the first time the discovery of an LCA derivative that effectively inhibits cell growth at submicromolar concentrations. We also propose that this activity is a result of a direct inhibition of USP2a deubiquitinase, leading to a significant decrease of the level of cyclin D1, a crucial regulator of cell-cycle progression. Our discovery shows that LCA derivatives are promising future therapeutics for the treatment of cyclin D1-dependent cancers. This study constitutes a starting point for the design of USP inhibitors based on an LCA scaffold to provide novel opportunities for cancer treatment.

STAR★METHODS

Detailed methods are provided in the online version of this paper and include the following:

- KEY RESOURCES TABLE
- CONTACT FOR REAGENT AND RESOURCE SHARING
- EXPERIMENTAL MODEL AND SUBJECT DETAILS
  - Cell Lines
- METHOD DETAILS
  - Cell Viability MTT Assay
  - Membrane Integrity LDH Release Assay

- ○ Cell Cycle Analysis
- ○ Caspase 3/7 Activity
- ○ Western Blotting
- ○ Colony Formation Assay
- ○ siRNA Transfection
- ○ RT PCR
- ○ USP2a Expression and Purification
- ○ Ubiquitin Expression and Purification
- ○ USP7 Expression and Purification
- ○ Ub-AMC and Di-Ub K63-2 Hydrolysis Assays
- ○ Thermal Shift Assay (TSA)
- ○ MALDI TOF Based High Throughput DUB Activity Assay
- ○ Compound Synthesis and Characterization
- ● QUANTIFICATION AND STATISTICAL ANALYSIS
- ● DATA AND SOFTWARE AVAILABILITY
- ● ADDITIONAL RESOURCES

SUPPLEMENTAL INFORMATION

Supplemental Information includes five figures and one table and can be found with this article online at http://dx.doi.org/10.1016/j.chembiol.2017.03.002.

AUTHOR CONTRIBUTIONS

K.M., L.S., G.D., and T.A.H designed the research. K.M. and L.S. performed the experiments. M. Tomala and M.L. synthesized the compounds. V.D.C. and M. Trost performed the high-throughput DUB activity assay. B.J.Z. provided the support with recombinant protein preparation. N.K.-T. contributed to the flow cytometry measurements. K.K. performed NMR experiments. T.A.H., G.D., K.M., and L.S. analyzed data and wrote the final version of the manuscript. All authors discussed the experiments and commented on the manuscript.

ACKNOWLEDGMENTS

This research has been supported by the UMO-2011/01/B/ST5/01744 grant from the National Science Centre, Poland, by the Project operated within the Foundation for Polish Science TEAM Program, co-financed by the EU European Regional Development Fund; and by a Marie Curie FP7-Reintegration-Grant within the seventh European Community Framework Program (to T.A.H.). The research was carried out with the equipment purchased from the European Regional Development Fund in the framework of the Polish Innovation Economy Operational Program (contract no. POIG.02.01.00-12-023/08). This work was funded by Medical Research Council UK (MC_UU_12016/5 to M.T.) and the pharmaceutical companies supporting the Division of Signal Transduction Therapy (DSTT) (AstraZeneca, Boehringer-Ingelheim, GlaxoSmithKline, Janssen Pharmaceutica, Merck KGaA, and Pfizer). We would like to thank the DNA cloning and Protein Production teams of the MRC Protein Phosphorylation and Ubiquitylation Unit as well as Dr. Axel Knebel for their support.

Received: February 19, 2016
Revised: September 26, 2016
Accepted: March 1, 2017
Published: March 23, 2017

REFERENCES

Alao, J.P. (2007). The regulation of cyclin D1 degradation: roles in cancer development and the potential for therapeutic invention. Mol. Cancer *6*, 24.

Bartkova, J., Lukas, J., Strauss, M., and Bartek, J. (1995). Cyclin D1 oncoprotein aberrantly accumulates in malignancies of diverse histogenesis. Oncogene *10*, 775–778.

Beers, E.P., and Callis, J. (1993). Utility of polyhistidine-tagged ubiquitin in the purification of ubiquitin-protein conjugates and as an affinity ligand for the purification of ubiquitin-specific hydrolases. J. Biol. Chem. *268*, 21645–21649.

Bernstein, C., Holubec, H., Bhattacharyya, A.K., Nguyen, H., Payne, C.M., Zaitlin, B., and Bernstein, H. (2011). Carcinogenicity of deoxycholate, a secondary bile acid. Arch. Toxicol. *85*, 863–871.

Chambard, J.-C., Lefloch, R., Pouysségur, J., and Lenormand, P. (2007). ERK implication in cell cycle regulation. Biochim. Biophys. Acta *1773*, 1299–1310.

Cheng, M., Sexl, V., Sherr, C.J., and Roussel, M.F. (1998). Assembly of cyclin D-dependent kinase and titration of p27Kip1 regulated by mitogen-activated protein kinase kinase (MEK1). Proc. Natl. Acad. Sci. USA *95*, 1091–1096.

Coverley, D., Laman, H., and Laskey, R.A. (2002). Distinct roles for cyclins E and A during DNA replication complex assembly and activation. Nat. Cell Biol. *4*, 523–528.

Davis, M.I., Pragani, R., Fox, J.T., Shen, M., Parmar, K., Gaudiano, E.F., Liu, L., Tanega, C., McGee, L., Hall, M.D., et al. (2016). Small molecule inhibition of the ubiquitin-specific protease USP2 accelerates cyclin D1 degradation and leads to cell cycle arrest in colorectal cancer and mantle cell lymphoma models. J. Biol. Chem. *291*, 24628–24640.

Diehl, J.A., Zindy, F., and Sherr, C.J. (1997). Inhibition of cyclin D1 phosphorylation on threonine-286 prevents its rapid degradation via the ubiquitin-proteasome pathway. Genes Dev. *11*, 957–972.

Diehl, J.A., Cheng, M., Roussel, M.F., and Sherr, C.J. (1998). Glycogen synthase kinase-3beta regulates cyclin D1 proteolysis and subcellular localization. Genes Dev. *12*, 3499–3511.

Farhana, L., Nangia-Makker, P., Arbit, E., Shango, K., Sarkar, S., Mahmud, H., Hadden, T., Yu, Y., and Majumdar, A.P.N. (2016). Bile acid: a potential inducer of colon cancer stem cells. Stem Cell Res. Ther. *7*, 181.

Fukami-Kobayashi, J., and Mitsui, Y. (1999). Cyclin D1 inhibits cell proliferation through binding to PCNA and cdk2. Exp. Cell Res. *246*, 338–347.

Goldberg, A.A., Beach, A., Davies, G.F., Harkness, T.A., Leblanc, A., and Titorenko, V.I. (2011). Lithocholic bile acid selectively kills neuroblastoma cells, while sparing normal neuronal cells. Oncotarget *2*, 761–782.

Grillo, M., Bott, M.J., Khandke, N., McGinnis, J.P., Miranda, M., Meyyappan, M., Rosfjord, E.C., and Rabindran, S.K. (2006). Validation of cyclin D1/CDK4 as an anticancer drug target in MCF-7 breast cancer cells: effect of regulated overexpression of cyclin D1 and siRNA-mediated inhibition of endogenous cyclin D1 and CDK4 expression. Breast Cancer Res. Treat. *95*, 185–194.

Hofmann, A.F. (2004). Detoxification of lithocholic acid, a toxic bile acid: relevance to drug hepatotoxicity. Drug Metab. Rev. *36*, 703–722.

Houten, S.M., Watanabe, M., and Auwerx, J. (2006). Endocrine functions of bile acids. EMBO J. *25*, 1419–1425.

Issaenko, O.A., and Amerik, A.Y. (2012). Chalcone-based small-molecule inhibitors attenuate malignant phenotype via targeting deubiquitinating enzymes. Cell Cycle *11*, 1804–1817.

Jackson, P.K., Chevalier, S., Philippe, M., and Kirschner, M.W. (1995). Early events in DNA replication require cyclin E and are blocked by p21CIP1. J. Cell Biol. *130*, 755–769.

Katona, B.W., Anant, S., Covey, D.F., and Stenson, W.F. (2009). Characterization of enantiomeric bile acid-induced apoptosis in colon cancer cell lines. J. Biol. Chem. *284*, 3354–3364.

Kim, J., Kim, W.-J., Liu, Z., Loda, M., and Freeman, M.R. (2012). The ubiquitin-specific protease USP2a enhances tumor progression by targeting cyclin A1 in bladder cancer. Cell Cycle *11*, 1123–1130.

Májer, F., Sharma, R., Mullins, C., Keogh, L., Phipps, S., Duggan, S., Kelleher, D., Keely, S., Long, A., Radics, G., et al. (2014). New highly toxic bile acids derived from deoxycholic acid, chenodeoxycholic acid and lithocholic acid. Bioorg. Med. Chem. *22*, 256–268.

Matulis, D., Kranz, J.K., Salemme, F.R., and Todd, M.J. (2005). Thermodynamic stability of carbonic anhydrase: measurements of binding affinity and stoichiometry using ThermoFluor. Biochemistry *44*, 5258–5266.

Musgrove, E.A., Caldon, C.E., Barraclough, J., Stone, A., and Sutherland, R.L. (2011). Cyclin D as a therapeutic target in cancer. Nat. Rev. Cancer *11*, 558–572.

Nagengast, F.M., Grubben, M.J., and van Munster, I.P. (1995). Role of bile acids in colorectal carcinogenesis. Eur. J. Cancer *31A*, 1067–1070.

Nicholson, B., Leach, C.A., Goldenberg, S.J., Francis, D.M., Kodrasov, M.P., Tian, X., Shanks, J., Sterner, D.E., Bernal, A., Mattern, M.R., et al. (2008). Characterization of ubiquitin and ubiquitin-like-protein isopeptidase activities. Protein Sci. *17*, 1035–1043.

Pantoliano, M.W., Petrella, E.C., Kwasnoski, J.D., Lobanov, V.S., Myslik, J., Graf, E., Carver, T., Asel, E., Springer, B.A., Lane, P., et al. (2001). High-density miniaturized thermal shift assays as a general strategy for drug discovery. J. Biomol. Screen *6*, 429–440.

Payne, C.M., Crowley-Weber, C.L., Dvorak, K., Bernstein, C., Bernstein, H., Holubec, H., Crowley, C., and Garewal, H. (2005). Mitochondrial perturbation attenuates bile acid-induced cytotoxicity. Cell Biol. Toxicol. *21*, 215–231.

Prasad, A.R., Prasad, S., Nguyen, H., Facista, A., Lewis, C., Zaitlin, B., Bernstein, H., and Bernstein, C. (2014). Novel diet-related mouse model of colon cancer parallels human colon cancer. World J. Gastrointest. Oncol. *6*, 225–243.

Renatus, M., Parrado, S.G., D'Arcy, A., Eidhoff, U., Gerhartz, B., Hassiepen, U., Pierrat, B., Riedl, R., Vinzenz, D., Worpenberg, S., et al. (2006). Structural basis of ubiquitin recognition by the deubiquitinating protease USP2. Structure *14*, 1293–1302.

Ritorto, M.S., Ewan, R., Perez-Oliva, A.B., Knebel, A., Buhrlage, S.J., Wightman, M., Kelly, S.M., Wood, N.T., Virdee, S., Gray, N.S., et al. (2014). Screening of DUB activity and specificity by MALDI-TOF mass spectrometry. Nat. Commun. *5*, 4763.

Sa, G., Guo, Y., and Stacey, D.W. (2005). The regulation of S phase initiation by p27Kip1 in NIH3T3 cells. Cell Cycle *4*, 618–627.

Schneider, C.A., Rasband, W.S., and Eliceiri, K.W. (2012). NIH Image to ImageJ: 25 years of image analysis. Nat. Methods *9*, 671–675.

Shan, J., Zhao, W., and Gu, W. (2009). Suppression of cancer cell growth by promoting cyclin D1 degradation. Mol. Cell *36*, 469–476.

Sharma, R., Majer, F., Peta, V.K., Wang, J., Keaveney, R., Kelleher, D., Long, A., and Gilmer, J.F. (2010). Bile acid toxicity structure-activity relationships: correlations between cell viability and lipophilicity in a panel of new and known bile acids using an oesophageal cell line (HET-1A). Bioorg. Med. Chem. *18*, 6886–6895.

Sherr, C.J., and Roberts, J.M. (2004). Living with or without cyclins and cyclin-dependent kinases. Genes Dev. *18*, 2699–2711.

Shi, Y., Solomon, L.R., Pereda-Lopez, A., Giranda, V.L., Luo, Y., Johnson, E.F., Shoemaker, A.R., Leverson, J., and Liu, X. (2011). Ubiquitin-specific cysteine protease 2a (USP2a) regulates the stability of Aurora-A. J. Biol. Chem. *286*, 38960–38968.

Shimura, T., Noma, N., Oikawa, T., Ochiai, Y., Kakuda, S., Kuwahara, Y., Takai, Y., Takahashi, A., and Fukumoto, M. (2012). Activation of the AKT/cyclin D1/Cdk4 survival signaling pathway in radioresistant cancer stem cells. Oncogenesis *1*, e12.

Singh, M., Bansal, S., Kundu, S., Bhargava, P., Singh, A., Motiani, R.K., Shyam, R., Sreekanth, V., Sengupta, S., and Bajaj, A. (2015). Synthesis, structure-activity relationship, and mechanistic investigation of lithocholic acid amphiphiles for colon cancer therapy. Medchemcomm *6*, 192–201.

Stacey, D.W. (2010). Three observations that have changed our understanding of cyclin D1 and p27 in cell cycle control. Genes Cancer *1*, 1189–1199.

Staels, B., and Fonseca, V.A. (2009). Bile acids and metabolic regulation: mechanisms and clinical responses to bile acid sequestration. Diabetes Care *32* (*Suppl 2*), S237–S245.

Tari, A.M., and Lopez-Berestein, G. (2000). Serum predominantly activates MAPK and akt kinases in EGFR- and ErbB2-over-expressing cells, respectively. Int. J. Cancer *86*, 295–297.

van Kuppeveld, F.J., van der Logt, J.T., Angulo, A.F., van Zoest, M.J., Quint, W.G., Niesters, H.G., Galama, J.M., and Melchers, W.J. (1992). Genus- and species-specific identification of mycoplasmas by 16S rRNA amplification. Appl. Environ. Microbiol. *58*, 2606–2615.

Vogel, S.M., Bauer, M.R., Joerger, A.C., Wilcken, R., Brandt, T., Veprintsev, D.B., Rutherford, T.J., Fersht, A.R., and Boeckler, F.M. (2012). Lithocholic acid is an endogenous inhibitor of MDM4 and MDM2. Proc. Natl. Acad. Sci. USA *109*, 16906–16910.

Wachs, F.-P., Krieg, R.C., Rodrigues, C.M.P., Messmann, H., Kullmann, F., Knüchel-Clarke, R., Schölmerich, J., Rogler, G., and Schlottmann, K. (2005). Bile salt-induced apoptosis in human colon cancer cell lines involves the mitochondrial transmembrane potential but not the CD95 (Fas/Apo-1) receptor. Int. J. Colorectal Dis. *20*, 103–113.

Yin, J.L., Shackel, N.A., Zekry, A., McGuinness, P.H., Richards, C., Putten, K.V., McCaughan, G.W., Eris, J.M., and Bishop, G.A. (2001). Real-time reverse transcriptase-polymerase chain reaction (RT-PCR) for measurement of cytokine and growth factor mRNA expression with fluorogenic probes or SYBR Green I. Immunol. Cell Biol. *79*, 213–221.

Yui, S., Saeki, T., Kanamoto, R., and Iwami, K. (2005). Characteristics of apoptosis in HCT116 colon cancer cells induced by deoxycholic acid. J. Biochem. *138*, 151–157.

Please cite this article as: Magiera et al., Lithocholic Acid Hydroxyamide Destabilizes Cyclin D1 and Induces G_0/G_1 Arrest by Inhibiting Deubiquitinase USP2a, Cell Chemical Biology (2017), http://dx.doi.org/10.1016/j.chembiol.2017.03.002

STAR★METHODS

KEY RESOURCES TABLE

REAGENT or RESOURCE	SOURCE	IDENTIFIER
Antibodies		
FITC-conjugated anti-BrdU antibody	BioLegend	Cat# 364104 RRID:AB_2564481
rabbit polyclonal anti-cyclin D1	Santa Cruz Biotechnology	Cat# sc-753 RRID:AB_2070433
mouse monoclonal anti-Cyclin D3	Cell Signaling Technology	Cat# 2936 RRID:AB_2070801
polyclonal anti-p53	Santa Cruz Biotechnology	Cat# sc-6243 RRID:AB_653753
rabbit monoclonal anti-p21	Cell Signaling Technology	Cat# 2947 RRID:AB_823586
rabbit monoclonal anti-p27	Cell Signaling Technology	Cat# 3686 RRID:AB_2077850
mouse monoclonal anti-Cyclin A2	Cell Signaling Technology	Cat# 4656 RRID:AB_2071958
mouse monoclonal anti-Cyclin E1	Cell Signaling Technology	Cat# 4129 RRID:AB_2071200
rabbit monoclonal anti-p-Akt(S473)	Cell Signaling Technology	Cat# 4060 RRID:AB_2315049
rabbit monoclonal anti-p-GSK-3β(S9)	Cell Signaling Technology	Cat# 5558 RRID:AB_10013750
mouse monoclonal anti-Aurora A	Santa Cruz Biotechnology	Cat# sc-373856 RRID:AB_10988868
rabbit polyclonal anti-cyclin A1	Santa Cruz Biotechnology	Cat# sc-7252 RRID:AB_1562274
rabbit monoclonal anti-GAPDH	Cell Signaling Technology	Cat# 2118 RRID:AB_561053
rabbit monoclonal anti-α-Tubulin	Cell Signaling Technology	Cat# 2125 RRID:AB_2619646
goat peroxidase-conjugated anti-rabbit	Cell Signaling Technology	Cat# 7074 RRID:AB_2099233
horse peroxidase-conjugated anti-mouse	Cell Signaling Technology	Cat# 7076 RRID:AB_330924
Bacterial and Virus Strains		
One Shot® BL21(DE3) Chemically Competent/E. coli/	Invitrogen (Thermo Fisher Scientific)	Cat#C600003
Chemicals, Peptides, and Recombinant Proteins		
THIAZOLYL BLUE TETRAZOLIUM BROMIDE, MTT	Sigma Aldrich	Cat#M5655-1G; CAS: 298-93-1
Dimethyl sulfoxide, DMSO, Hybri-Max (for cell treatment and cryopreservation)	Sigma Aldrich	Cat#D2650-5X10ML; CAS: 67-68-5
5-Bromo-2′-deoxyuridine (BrdU) BioUltra, ≥99%	Sigma Aldrich	Cat#B9285-50MG; CAS: 59-14-3
Staurosporine	Santa Cruz Biotechnology	Cat# sc-3510 A; CAS: 62996-74-1
Propidium Iodide (PI)	Serva	Cat#33671.01; CAS: 25535-16-4
Hoechst 33342	Thermo Fisher Scientific	Cat#62249; CAS: 23491-52-3
RIPA buffer	Sigma Aldrich	Cat#R0278-50ML
Protease inhibitor cocktail	Sigma Aldrich	Cat#P8340-5ML
Phosphatase inhibitor cocktail	Roche	Cat#04 906 845 001
Albumin, Bovine, Fraction V. Heat Shock Isolation (BSA)	BioShop	Cat#ALB001.250
Clarity Western ECL Substrate	BioRad	Cat#1705061
Lipofectamine 2000	Life Technologies	Cat#11668-027
Renozol	GenoPlast Biochemicals	Cat#BMGPB1100-2
M-MLV Reverse Transcriptase	Promega	Cat# M1701
Hydroxylamine solution 50 wt. % in H2O	Sigma Aldrich	Cat# 438227; CAS: 7803-49-8
Lithocholic acid	Sigma Aldrich	Cat# L6250; CAS: 434-13-9
Glycine	Sigma Aldrich	Cat# 410225; CAS: 56-40-6
Ammonium hydroxide solution	Sigma Aldrich	Cat# 09859; CAS: 13550-49-7
4-(2-Aminoethyl)morpholine	Sigma Aldrich	Cat# A55004; CAS: 2038-03-1
Acetyl chloride	Sigma Aldrich	Cat# 320129; CAS: 75-36-5
Chromium(VI) oxide	Sigma Aldrich	Cat# 675644; CAS: 1333-82-0
Morpholine	Sigma Aldrich	Cat# 394467; CAS: 110-91-8
Isobutyl chloroformate	Sigma Aldrich	Cat# 177989; CAS: 543-27-1

(*Continued on next page*)

Continued

REAGENT or RESOURCE	SOURCE	IDENTIFIER
DIC	Sigma Aldrich	Cat# D125407; CAS: 693-13-0
Chloroform-d	Armar	Cat# 013300.2040; CAS: 865-49-6
DMSO-d6	Armar	Cat# 015600.2035; CAS: 2206-27-1
Sulfuric acid	POCH	Cat# 575000115; CAS: 7664-93-9
Acetic acid	POCH	Cat# 568760421; CAS: 64-19-7
Methanol	POCH	Cat# 621990426; CAS: 67-56-1
Dichloromethane	POCH	Cat# 628410421; CAS: 75-09-2
TEA	POCH	Cat# 848930423; CAS: 121-44-8
Acetonitrile	POCH	Cat# 102640111; CAS: 75-05-8
Sodium Bicarbonate	POCH	Cat# 810530115; CAS: 144-55-8
Sodium Sulfate Anhydrous	POCH	Cat# 807870111; CAS: 7727-73-3
LB BROTH (MILLER)	BioShop	Cat#LBL407.5
IPTG , Reagent Grade, min 99%	BioShop	Cat#IPT002.25; CAS: 367-93-1
Phenylmethanesulfonyl fluoride (PMSF)	Sigma Aldrich	Cat#P7626; CAS: 329-98-6
Chelating Sepharose Fast Flow	GE Healthcare	Cat#17057502
Q Sepharose Fast Flow	GE Healthcare	Cat#17051001
Ubiquitin-AMC	VIVA Bioscience	Cat#VB2906-0050
Di-Ubiquitin K63-2	LifeSensors	Cat#DU6302
NSC-632839	LifeSensors	Cat#SI9689; CAS: 157654-67-6
SYPRO® Orange Protein Gel Stain	Thermo Fisher Scientific	Cat#S6650
vector pGEX-6p-1	GE Healthcare	Cat#28-9546-48
PreScission Protease	GE Healthcare	Cat# 27-0843-01
Recombinant USP2 (258-605)	This paper	N/A
Recombinant Ubiquitin (1-76)	This paper	N/A
Recombinant USP7 (208-561)	This paper	N/A
DUB enzymes and ubiquitin for MALDI TOF High Throughput DUB Activity Assay	(Ritorto et al., 2014)	N/A
**Critical Commercial Assays**		
CytoTox-ONE™ Homogeneous Membrane Integrity Assay	Promega	Cat# G7891
Caspase-Glo® 3/7 Assay	Promega	Cat# G8091
GoTaq qPCR Master Mix	Promega	Cat# A6001
**Experimental Models: Cell Lines**		
Human: HCT116	ECACC	Cat# 91091005, RRID:CVCL_0291
Human: U-2 OS	ECACC	Cat# 92022711, RRID:CVCL_0042
Human: SAOS-2	ECACC	Cat# 89050205, RRID:CVCL_0548
Human: MCF-7	ECACC	Cat# 86012803, RRID:CVCL_0031
**Oligonucleotides**		
Human cyclin D1 siRNA	Santa Cruz Biotechnology	Cat# sc-29286
siRNA-A	Santa Cruz Biotechnology	Cat# sc-37007
siRNA-B	Santa Cruz Biotechnology	Cat# sc-44230
siRNA-C	Santa Cruz Biotechnology	Cat# sc-44231
Oligo-dT: TTTTTTTTTTTTTTT	Genomed	N/A
RT PCR CCND1 mRNA primer 1: TGCCAACCTCCTCAACGACCG	This paper	N/A
RT PCR CCND1 mRNA primer 2: TCGCAGACCTCCAGCATCCAG	This paper	N/A
RT PCR GAPDH mRNA primer 1: TGCACCACCAACTGCTTAGC	Genomed ; (Yin et al., 2001)	N/A

(*Continued on next page*)

Continued

REAGENT or RESOURCE	SOURCE	IDENTIFIER
RT PCR GAPDH mRNA primer 2: GGCATGGACTGTGGTCATGAG	Genomed; (Yin et al., 2001)	N/A
Mycoplasma detection primer 1: GPO-1: ACTCCTACGGGAGGCAGCAGTA	Genomed; (van Kuppeveld et al., 1992)	N/A
Mycoplasma detection primer 2: MGSO: TGCACCATCTGTCACTCTGTTAACCTC	Genomed; (van Kuppeveld et al., 1992)	N/A
USP7 (208-561) primer forward: GCTACTCGAGTTACTATTCCTGCCGCTCC	Genomed	N/A
USP7 (208-561) primer reverse: GCAAGGATCCAAGAAGCACACAGGCTAC	Genomed	N/A
Recombinant DNA		
pet16b-UB (1-76, human)	Prof. Stefan Jentsch, Max Planck Institute for Biochemistry, Munich, Germany; (Beers and Callis, 1993)	N/A
pet24a-USP2 (258-605, human)	Prof. Tad Holak, Max Planck Institute for Biochemistry, Munich, Germany; (Renatus et al., 2006)	N/A
human USP7 ORF	BioScience	OCABo5050A1122D
pGEX-6p-1-USP7 (208-561, human)	This paper	N/A
Software and Algorithms		
ModFit LT Software v4.1.7	Verity Software House	http://www.vsh.com/products/mflt/index.asp
Image Lab v5.0	BioRad	http://www.bio-rad.com/en-us/product/image-lab-software
ImageJ 1.48v	Schneider et al., 2012	https://imagej.nih.gov/ij/

CONTACT FOR REAGENT AND RESOURCE SHARING

Further information and requests for resources and reagents should be directed to and will be fulfilled by the Lead Contact, Lukasz Skalniak (lukasz.skalniak@uj.edu.pl).

EXPERIMENTAL MODEL AND SUBJECT DETAILS

Cell Lines

Human colorectal carcinoma cell lines HCT116 p53wt and HCT116 p53-/-, and human osteosarcoma cell lines U-2 OS and SAOS-2 were cultured in Mc Coy's medium supplemented with 10% Fetal Bovine Serum (FBS) (BioWest). Human breast adenocarcinoma cell line (female) MCF-7 was cultured in DMEM medium supplemented with 10% FBS.

The cell lines were routinely verified for mycoplasma infection using a PCR-based method (van Kuppeveld et al., 1992).

METHOD DETAILS

Cell Viability MTT Assay

For MTT assay, the cells were seeded on 96-well transparent plates and treated the next day with the compounds or DMSO for 6 days. Thiazolyl Blue Tetrazolium Bromide (MTT, Sigma Aldrich) was added for 60 minutes to a final concentration of 500 ng/ml. The medium was removed and MTT crystals were dissolved in isopropanol supplemented with 40 mM HCl. The absorbance was measured with Infinite 200 microplate reader (Tecan Group Ltd.) at 570 nm with the reference wavelength 650 nm for background subtraction.

Membrane Integrity LDH Release Assay

Membrane integrity LDH release assay was performed using CytoTox-ONE kit (Promega). The cells were seeded on 96-well black plates, and after 24 hours treated with the compounds or DMSO for 2 days. The assay was performed according to the manufacturer's instructions. For each well the data was calculated as a % of total LDH content, measured for the lysed cells, treated in the same manner as cells on that well.

Cell Cycle Analysis

The cells were treated with DMSO or LCAHA for 48 hours and pulse-labelled with 10 μM bromodeoxyuridine (BrdU, Sigma Aldrich) for the last hour of the treatment. After that, the cells were either harvested by trypsinization and fixed with 96% ethanol, or cultured for additional 3-9 hours in the absence of BrdU before harvesting and fixation. The cells were stained with PI and FITC-conjugated anti-BrdU antibody (BioLegend, cat. 364104) and analysed with Fortessa flow cytometer (Becton Dickinson). Cell cycle distribution was analysed using ModFit LT Software (Verity Software House).

Caspase 3/7 Activity

The cells were plated on the 96-well white, flat bottom plates (Falcon) and treated with DMSO, LCAHA or Staurosporine. For the detection of caspase activity Caspase-Glo 3/7 Assay System was used (Promega) according to the manufacturer's instructions. Due to the observed growth-inhibitory properties of LCAHA the results were normalized to cell numbers, calculated from the pictures of Hoechst-stained cells seeded on transparent culture plates and treated identically as the cells seeded for caspase activity assay (mean nuclei number per picture was 6452 for 3 days treatment with DMSO, and 3957, 4229 and 2783 for the cells treated with increasing concentrations of LCAHA).

Western Blotting

Total cell lysates were prepared with RIPA buffer (Sigma Aldrich) containing protease inhibitor cocktail (Sigma Aldrich) alone or with phosphatase inhibitor cocktail (Roche). Following the electrophoresis and transfer, PVDF membranes were blocked with 4% BSA (BioShop) in TBS-N buffer and incubated with primary antibody at 4°C overnight. After four washes, the addition of secondary antibodies and additional four washes, the detection was performed using Clarity Western ECL Substrate (BioRad) and ChemiDoc MP system (BioRad). The densitometry analysis was performed with Image Lab software (BioRad). All measured values, besides cyclin D1 stability experiment, were normalized to GAPDH or α-tubulin expression level.

The following antibodies and dilutions were used: rabbit polyclonal anti-cyclin D1 (1:200, Santa Cruz Biotechnology (SCBt), cat. sc-753), mouse monoclonal anti-Cyclin D3 (1:1 000, Cell Signaling Technology (CST), cat. 2936), polyclonal anti-p53 (1:200, SCBt, cat. sc-6243), rabbit monoclonal anti-p21 (1:1 000, CST, cat. 2947), rabbit monoclonal anti-p27 (1:1 000, CST, cat. 3686), mouse monoclonal anti-Cyclin A2 (1:1 000, CST, cat. 4656), mouse monoclonal anti-Cyclin E1 (1:1 000, CST, cat. 4129), rabbit monoclonal anti-p-Akt(S473) (1:1 000, CST, cat. 4060), rabbit monoclonal anti-p-GSK-3β(S9) (1:1 000, CST, cat. 5558), mouse monoclonal anti-Aurora A (1:100, SCBt, cat. sc-373856), rabbit polyclonal anti-cyclin A1 (1:200, SCBt, cat. sc-7252), rabbit monoclonal anti-GAPDH (1:4 000, CST, cat. 2118), rabbit monoclonal anti-α-Tubulin (1:4 000, CST, cat. 2125), goat peroxidase-conjugated anti-rabbit (1:2 000, CST, cat. 7074), horse peroxidase-conjugated anti-mouse (1:2 000, Cell Signaling, cat. 7076).

Colony Formation Assay

For the colony formation assay MCF-7 or SAOS-2 cells were treated with DMSO or 5 μM LCAHA for 5 days. One thousand of the cells was then seeded on 6-well plates and cultured for 2-4 weeks until the generation of well-visible clones. The plates were stained with crystal violet, imaged with the ChemiDoc MP system and analyzed using the ImageJ software (Schneider et al., 2012) and 'Analyze particles' tool. The surviving fraction (SF) was calculated using the equation: $SF = (PE_{\text{of treated sample}} / PE_{\text{of control}}) * 100\%$, where PE (plating efficiency) = $N_{\text{colonies}} / N_{\text{cells plated}}$.

siRNA Transfection

HCT116 cells were seeded on 6-well plates and transfected the next day with Lipofectamine 2000 (Life Technologies) and 100 pmol of siRNA according to the manufacturer's instructions. No medium change was performed following the transfection. The following siRNA products were used: cyclin D1 siRNA (h): sc-29286 and the mixture of three negative control siRNA sequences: siRNA-A, siRNA-B and siRNA-C (Santa Cruz).

RT PCR

Total RNA was isolated with Renozol (GenoPlast Biochemicals) according to the manufacturer's instructions. Five hundred ng of RNA was reverse-transcribed using M-MLV RT (Promega) and oligo-dT primer. RT PCR was performed with the use of GoTaq qPCR Master Mix (Promega) and the following primers: for *CCND1* mRNA: TGCCAACCTCCTCAACGACCG and TCGCAGACCTCCAG CATCCAG, for *GAPDH* mRNA: TGCACCACCAACTGCTTAGC and GGCATGGACTGTGGTCATGAG (Yin et al., 2001). The hybridization step was carried out at 60°C and the program involved 30 amplification cycles. The products were separated on 1% agarose gel and visualized with ChemiDoc MP system.

USP2a Expression and Purification

Human USP2a (residues 258-605) was expressed in the Escherichia coli BL21 (DE3, Invitrogen). Cells were grown in LB medium containing 100 μg/ml ampicillin at 37 °C and induced with 0.5 mM IPTG at OD_{600} of 0.7-0.9 and cultured for additional 5 h at 37 °C. Cells

were harvested by centrifugation and frozen at -20 °C. USP2a purification was carried out according to optimized protocol (Renatus et al., 2006). In brief, cells from 6 liters culture were resuspended in 300 ml of lysis buffer (10 mM Tris/HCl pH=8.0, 1 mM $MgCl_2$, 5 mM β-mercapthoetanol, 10 μM PMSF) and ruptured by sonication. After centrifugation supernatant was loaded on a Chelating Sepharose Fast Flow (GE Healthcare) charged with nickel ions. The column was washed with lysis buffer and protein was eluted with lysis buffer supplemented with 250 mM imidazole. Fractions containing USP2a were combined and further purified on Q-Sepharose Fast Flow (GE Healthcare) column. USP2 protein was in the flow-through fraction.

The last purification step consisted of size exclusion chromatography on HiLoad 16/60 Superdex 75 prep grade (GE Healthcare) in PBS pH=7.4 containing 5 mM DTT. USP2a was stored for further experiments as 0.01 mM protein stock with 10% glycerol at -80 °C.

Ubiquitin Expression and Purification

Escherichia coli BL21 (DE3, Invitrogen) was transformed with pet16b-UBwt (1-76, human) and grown in LB medium containing 100 μg/ml ampicillin at 37 °C. Protein expression was induced with 1 mM IPTG at OD_{600} of 0.7-0.9 and cultured for additional 6 h at 37 °C. Cells were harvested by centrifugation and frozen at -20 °C. Ubiquitin purification was carried out according to optimized protocol (Beers and Callis, 1993). In brief, cells from 4 liters culture were resuspended in 200 ml of lysis buffer (50 mM NaH2PO4 pH=8.0, 300 mM NaCl, 1 mM imidazole) containing 1 mg/ml lysozyme. After incubation of cells on ice (10 min), NaCl and PMSF were added to final concentrations 600 mM and 2 mM, respectively. Cells were raptured by sonication. Cleared supernatant was loaded on a Chelating Sepharose Fast Flow (GE Healthcare) charged with nickel ions. The column was subsequently washed with lysis buffer, lysis buffer without NaCl, lysis buffer adjusted to pH=5.5 and pH=4.5. Protein was eluted with lysis buffer supplemented with 250 mM imidazole.

As the last purification step size exclusion chromatography on HiLoad 16/60 Superdex 75 prep grade (GE Healthcare) equilibrated with PBS pH=7.4 was used. Ubiquitin was stored for further experiments at 0.5-2 mM concentration at -20 °C.

USP7 Expression and Purification

Human USP7 catalytic domain (residues 208-561) was cloned into the pGEX-6P-1 vector (GE Healthcare) and expressed in the E. coli BL21 (DE3, Invitrogen). Cells were grown in LB medium containing 100 μg/ml ampicillin at 37 °C and induced with 0.5 mM IPTG at OD_{600} of 0.7-0.9 and cultured overnight at 16 °C. Cells were harvested by centrifugation. Next cells were resuspended in buffer A (50 mM Tris/HCl pH 9.0, 50 mM NaCl, 3 mM β-mercapthoetanol) and disrupted by sonication. Cell lysate were clarified by centrifugation (45 000 g, 40 min.) and dialyzed against buffer A. Supernatant was loaded onto Q-Sepharose column and proteins were eluted with NaCl gradient. Fractions containing GST fused USP7 were combined, concentrated and dialyzed against buffer (50 mM Tris/HCl pH 7.0, 50 mM NaCl, 3 mM β-mercapthoetanol). During dialysis protein was digested with PreScission Protease (GE Healthcare). USP7 protein and cleaved GST were separated on Mono Q HR 10/10 column (GE Healthcare). The last purification step consisted of size exclusion chromatography on HiLoad 16/60 Superdex 75 prep grade (GE Healthcare) in 50 mM Tris/HCl, 150 mM NaCl, 2 mM DTT.

Ub-AMC and Di-Ub K63-2 Hydrolysis Assays

For ubiquitin substrate hydrolysis assays human recombinant USP2a catalytic domain (residues 258-605) was used. The assays were performed using Infinite 200 PRO – Tecan plate reader and 96-well, black Greiner microplates in a 100 μl reaction volume.

Ub-AMC-hydrolysis assay was performed in a reaction buffer (50 mM Tris/HCl, pH=7.5, 1 mM EDTA, 1 mM $MgCl_2$). USP2a (10 nM) was preincubated with an excess of tested compound for 30 minutes and fluorescence changes were recorded at excitation and emission wavelengths of 355 nm and 460 nm respectively, immediately after Ub-AMC (500 nM, Viva Biosience) addition.

Di-Ub K63-2 hydrolysis assay was performed in 50 mM Tris/HCl pH=7.5, 1mM DTT. USP2a (20 nM) was preincubated with an excess of tested compound for 30 min. Fluorescence changes were recorded at excitation and emission wavelengths of 540 nm and 580 nm respectively, immediately after Di-Ub (200 nM, LifeSensors) addition.

The excess of the wild type ubiquitin or NSC 632839 was used in both assays as the inhibition control. All assays were performed simultaneously in triplicate and results are presented as mean values ± SD.

Thermal Shift Assay (TSA)

TSA analysis was carried out by monitoring the fluorescence of SYPRO Orange Dye (Life Technologies) in the presence of USP2a and tested compounds at increasing temperatures (form 22 to 98°C). Experiments were performed in PBS pH=7.4 buffer. USP2a (1 μM) was incubated alone and with LCAE or LCAHA compounds (both at 50 μM). Constant temperature gradient was applied (0.2°C/min) and fluorescence changes were monitored using real time thermocycler (BioRad). Melting temperature (Tm) was estimated from the first derivative of fluorescence intensity as a function of temperature.

MALDI TOF Based High Throughput DUB Activity Assay

The assay was performed as described before (Ritorto et al., 2014). Thirty one human DUBs were freshly diluted in reaction buffer (40mM Tris–HCl, pH 7.6, 5mM DTT, 0.005% BSA) to the proper concentrations. Enzymes were incubated with the LCAHA and

Please cite this article as: Magiera et al., Lithocholic Acid Hydroxyamide Destabilizes Cyclin D1 and Induces G_0/G_1 Arrest by Inhibiting Deubiquitinase USP2a, Cell Chemical Biology (2017), http://dx.doi.org/10.1016/j.chembiol.2017.03.002

LCAE at 100 μM final concentration for 30 min at room temperature. Diubiquitin topoisomers (K63, K48, K11 and M1) were diluted to 0.2 μl/μg and added to the reaction mixture using a Nanoliter pipetting system (Mosquito LabTech) to the final concentration of 1.5 μM. The plate was sealed and incubated for 30 min at room temperature and stopped by adding TFA to a final concentration of 2% (v/v). The terminated reaction was then transferred (1.050 μl) to a 384 plate (LSVD, TTP Labtech), spiked with ^{15}N-ubiquitin internal standard (0.15 μl, 16 μM) and mixed 1:1 with 2.5 DHAP matrix freshly prepared (7.6 mg of 2,5 DHAP in 375 ml ethanol and 125 ml of an aqueous 12 mg/ml diammonium hydrogen citrate). The resulting matrix/reaction mixture was spotted in 200 μl aliquots onto an MTP AnchorChip 1,536 TF (600 mm anchor, Bruker Daltonics).

Mass spectrometry data was acquired on an UltrafleXtreme MALDI-TOF mass spectrometer (Bruker Daltonics) with Compass 1.3 control and processing software. The sample carrier was taught before each analysis to optimize and centre laser shooting. Internal calibration was performed before each analysis using the ^{15}N-Ub peak $[M+H]^+$ average = 8,569.3). Samples were analyzed in automatic mode (AutoXecute, Bruker Daltonics) as previously reported (Ritorto et al., 2014). For area calculation, the complete isotopic distribution was taken into account. An in-house made script was used to report - ^{15}N and mono-ubiquitin areas; plotting of graphs, calculation of standard deviation and coefficient of variation (%) were processed in Microsoft Excel.

Compound Synthesis and Characterization

3-hydroxylithocholanamide (LCANH$_2$)

Lithocholic acid (200 mg, 0.53 mmol) was dissolved in DCM (7 ml) and TEA (0.3 ml). The solution was cooled to 0°C and isobuthyl chloroformate (190 μl, 1.45 mmol) was added dropwise. The mixture was stirred for 15 min at 0°C and cold 25% ammonium hydroxide was added slowly until the white precipitate stopped forming. The crude product was filtered off and dried under vacuum and crystalized from methanol to give (160 mg 0.43 mmol) (80% yield) as white needles, mp 216-219°C. IR (ATR): 3453, 3382, 3189, 2933, 2867, 1667 cm^{-1}. ^{1}H NMR (300 MHz, $CDCl_3$) δ = 0.61 (s, 3H, CH_3), 0.85-0.89 (m, 6H), 0.91-1.25 (m, 11H), 1.25-1.42 (m, 7H), 1.43-1.57 (m, 2H), 1.57-1.72 (m, 3H), 1.72-1.85 (m, 2H), 1.85-1.99 (m, 2H), 1.99-2.12 (m, 1H), 3.35-3.44 (m, 1H), 4.45 (d, *J* 4.45 Hz, 1H), 6.64 (s, 1H, NH), 7.22 (s, 1H, NH). ^{13}C NMR (75 MHz, $CDCl_3$) δ = 11.9, 18.3, 20.4, 23.3, 23.8, 26.2, 26.9, 27.7, 30.4, 31.4, 32.1, 34.2, 35.0, 35.2, 35.4, 36.3, 40.0, 41.5, 42.3, 55.6, 56.1, 69.9, 174.7 ($CONH_2$). HRMS (ESI) calcd for $C_{24}H_{41}NO_2$: 398.3035; found: 398.3030 $[M+Na]^+$.

^{1}H NMR of compound LCANH$_2$ (300 MHz, DMSO-d$_6$):

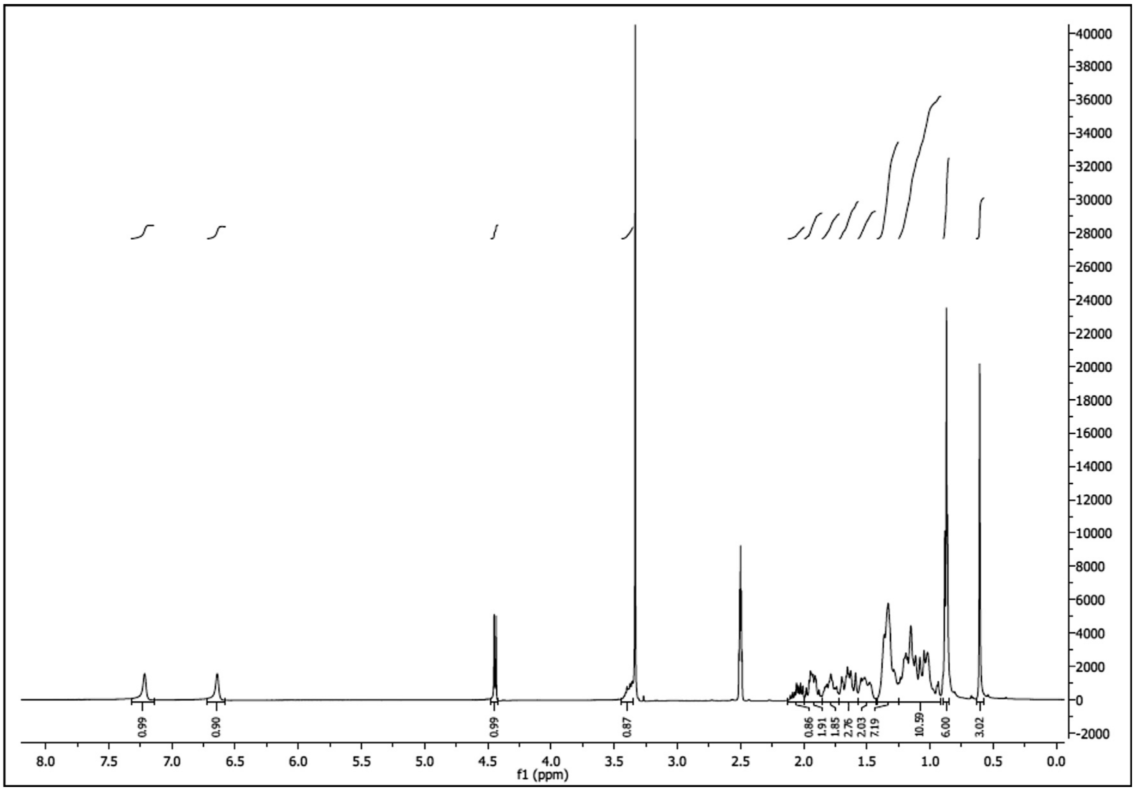

^{13}C NMR of compound $LCANH_2$ (75 MHz, DMSO-d_6):

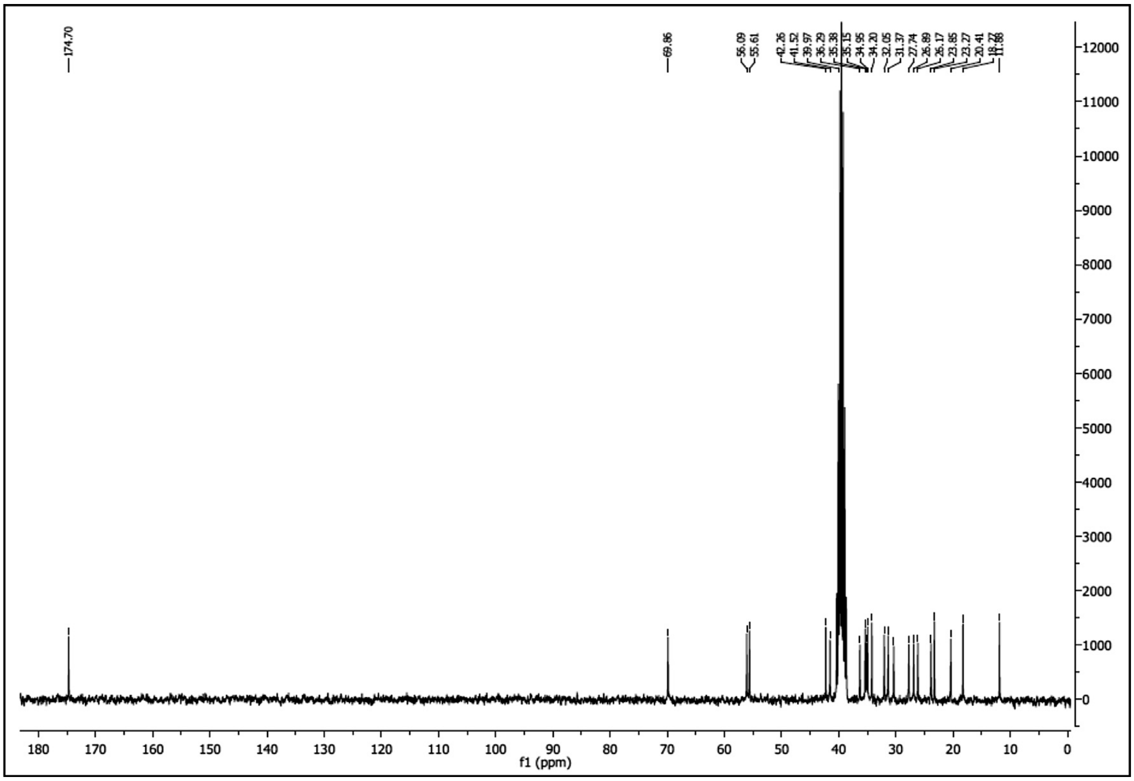

N-(3-hydroxylithocholanolyl)glycine (LCAGLY)

Lithocholic acid (500 mg, 1.33 mmol) and methyl glycinate hydrochloride (164 mg, 1.31 mmol) were dissolved in 3 ml of anhydrous DCM at 0°C and TEA (325 μl, 2.33 mmol) was added. The mixture was stirred for 15 minutes, after which DIC (271 μl, 1.38 mmol) was dropped. The reaction was left overnight at RT and then was diluted with 10 ml of DCM, washed with 1M HCl, brine and dried over anhydrous Na_2SO_4. Solvent was removed under reduced pressure and the resulting compound was purified by flash column chromatography (DCM) and recrystallized from ethanol/water as white needles. So obtained methyl N-(3-hydroxylithocholanolyl)glycinate was dissolved in 20% methanol/water solution of LiOH and stirred for an hour. Solvent was evaporated under reduced pressure. Water solution was acidified with conc. HCl and the resulting white precipitate was filtered, washed with distilled water and dried under vacuum. The compound was recrystallized from ethanol/water giving (403 mg, 0.93 mmol) of white solid (69% yield) mp. 178-182°C. IR (ATR): 3516, 3267, 2938, 2867, 1732, 1608, 1573, 1204 cm^{-1}. ^1H NMR (300 MHz, DMSO-d6) δ = 0.61 (s, 3H, CH_3), 0.86-0.90 (m, 6H), 0.93-1.24 (m, 11H), 1.25-1.43 (m, 8H), 1.44-1.87 (m, 8H), 1.88-1.97 (m, 1H), 1.96-2.08 (m, 1H), 2.09-2.21 (m, 1H), 3.70 (d, 2H *J* 5.9 Hz), 4.19-4.63 (m, 1H), 8.07 (t, 1H *J* 5.8 Hz). ^{13}C NMR (75 MHz, DMSO-d6) δ = 11.9, 18.3, 20.4, 23.2, 23.8, 26.1, 26.9, 27.7, 30.4, 31.4, 32.1, 34.2, 34.9, 35.1, 35.4, 36.3, 40.0, 41.5, 42.2, 55.6, 56.1, 69.8, 144.8, 171.9 (CO-NH), 172.6 (CO-OH). HRMS (ESI) calcd for $C_{26}H_{43}NO_4$: 456.3090; found: 456.3074 $[M+Na]^+$.

^{1}H NMR of compound LCAGLY (300 MHz, DMSO-d_6):

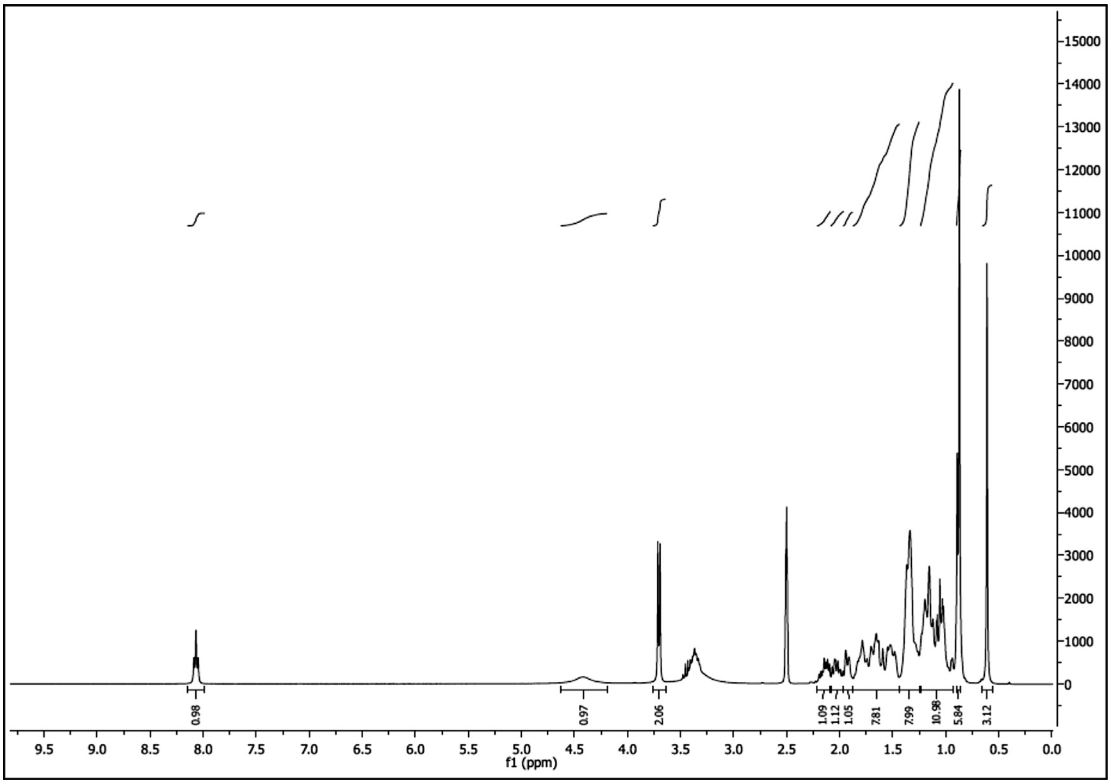

^{13}C NMR of compound LCAGLY (75 MHz, DMSO-d_6):

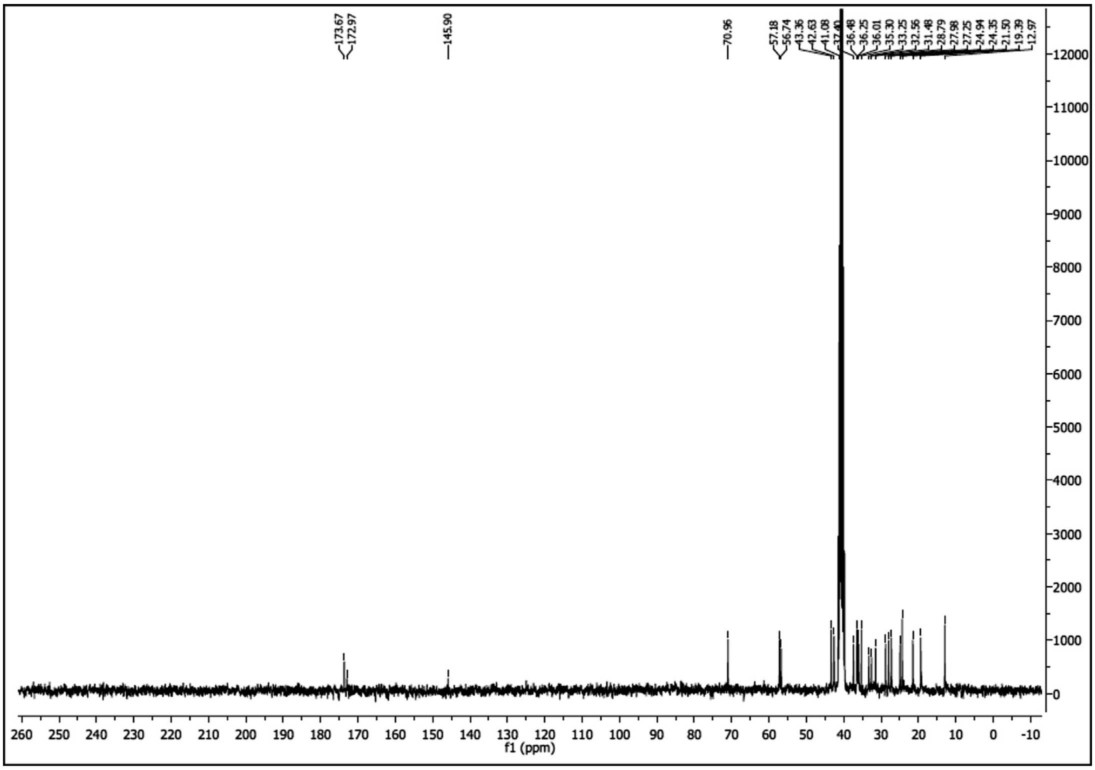

N-lithocholanolyl Morpholine (LCAMR)

To a suspension of lithocholic acid (100 mg, 0.26 mmol) and morpholine (21.25 μl, 0.24 mmol) in anhydrous DCM (3 ml) at 0°C, TEA (60 μl, 0.430 mmol) was added. The mixture was cooled to 0°C and DIC (25 μl, 0.13 mmol) was added. The mixture was stirred 15 minutes at 0°C, heated to RT and left overnight. The reaction was diluted with 10 ml of DCM and washed with 1M HCl, saturated $NaHCO_3$ and distilled water. The organic layer was dried over Na_2SO_4 and purified by flash column chromatography (DCM: AcOEt 10:1). Collected fractions were evaporated giving white solid (83 mg, 0.19 mmol) (70% yield) mp 133-135°C.IR (ATR): 3382, 2925, 2851, 1628, 1454 cm^{-1}. ^{1}H NMR (300 MHz, DMSO-d6) δ = 0.61 (s, 3H, CH_3), 0.86-0.91 (m, 6H), 0.92-1.25 (m, 11H), 1.26-1.40 (m, 7H), 1.44-1.88 (m, 7H), 1.89-1.96 (m, 1H), 2.11-2.25 (m, 1H), 2.25-2.40 (m, 1H), 3.35-3.44 (m, 5H), 3.48-3.60 (m, 4H), 4.38-4.46 (m, 1H). ^{13}C NMR (75 MHz, DMSO-d6) δ = 11.8, 18.3, 20.4, 23.2, 23.8, 26.1, 26.9, 27.7, 29.1, 30.4, 30.9, 34.2, 35.0, 35.1, 35.4, 36.3, 39.7, 40.0, 41.4, 41.5, 42.3, 45.4, 55.5, 56.0, 66.1, 69.8, 171.2 (CONH). HRMS (ESI) calcd for $C_{28}H_{47}NO_3$: 468.3454; found: 468.3437 $[M+Na]^+$.

^{1}H NMR of compound LCAMR (300 MHz, DMSO-d_6):

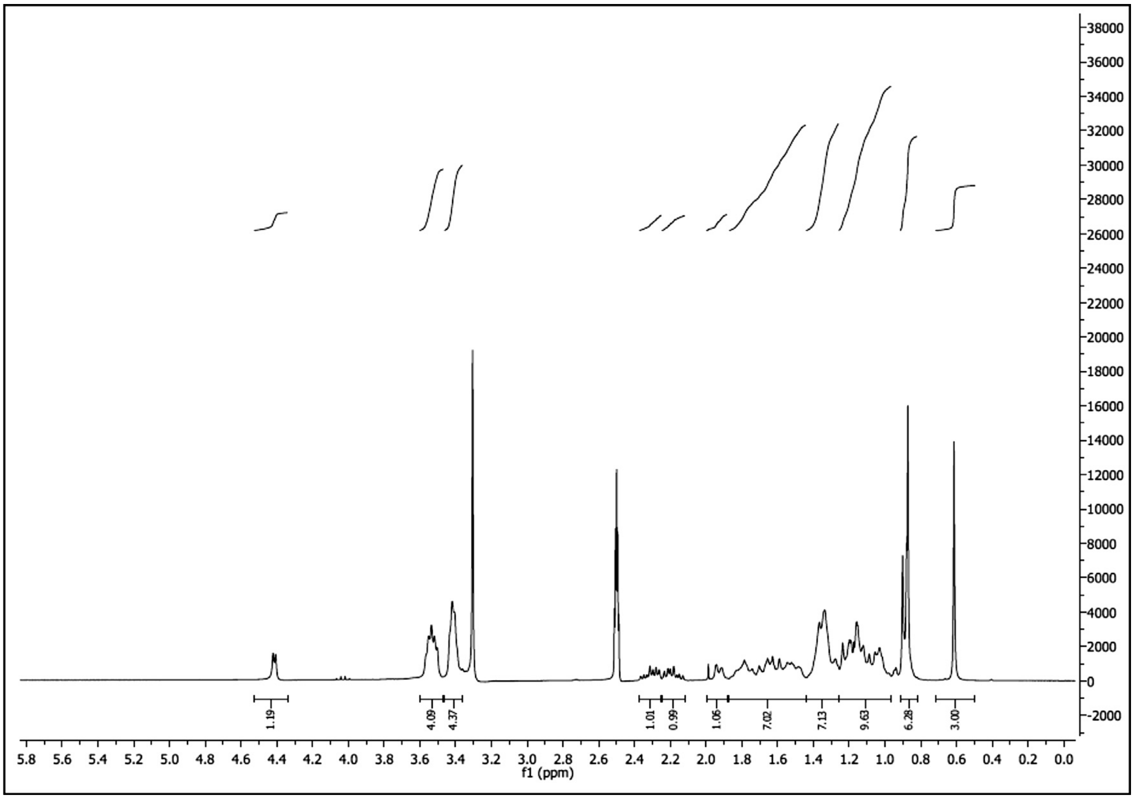

Please cite this article as: Magiera et al., Lithocholic Acid Hydroxyamide Destabilizes Cyclin D1 and Induces G_0/G_1 Arrest by Inhibiting Deubiquitinase USP2a, Cell Chemical Biology (2017), http://dx.doi.org/10.1016/j.chembiol.2017.03.002

^{13}C NMR of compound LCAMR (75 MHz, DMSO-d_6):

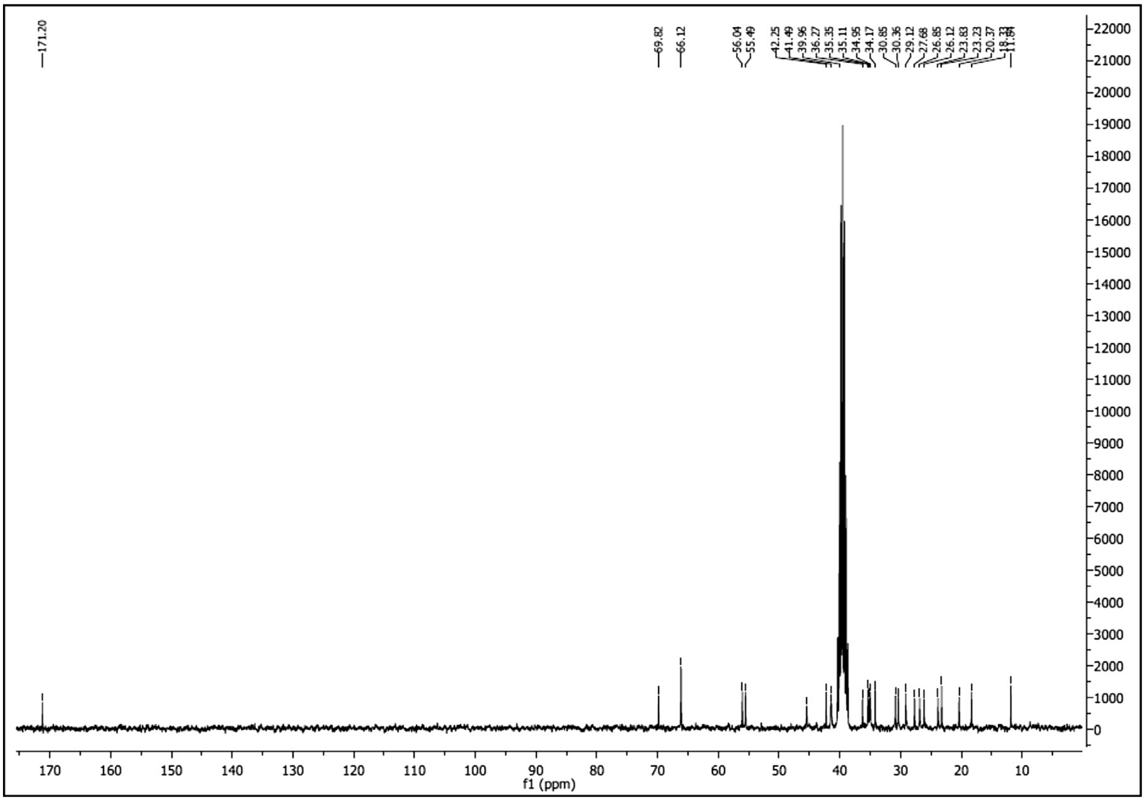

N-(cyanomethyl)-3-hydroxylithocholanamide (LCACN)

Lithocholic acid (100 mg, 0.26 mmol) and aminoacetonitrile hydrochloride (24 mg, 0.26mmol) were treated in the same way as in the synthesis of N-lithocholanolyl 4-(2-aminoethyl)morpholine. The product was purified by crystallization from n-hexan : AcOEt 2:1. As white crystals (77 mg, 0.19 mmol) (70% yield.) mp 180-183°C. IR (ATR): 3460, 3255, 2932, 2864, 1667 cm^{-1}. ^{1}H NMR (600 MHz, DMSO-d6) δ = 0.61 (s, 3H, CH_3), 0.86-0.88 (m, 6H), 0.89-0.93 (m, 1H), 0.96-1.11 (m, 5H), 1.12-1.25 (m, 6H), 1.31-1.39 (m, 6H), 1.47-1.55 (m, 2H), 1.57-1.64 (m, 1H), 1.65-1.70 (m, 2H), 1.75-1.83 (m, 2H), 1.90-1.94 (m, 1H), 2.01-2.07 (m, 1H), 2.13-2.18 (m, 1H), 2.38-2.40 (m, 1H), 4.08 (d, *J* 5.7 Hz, 2H, CH_2), 4.42 (s, 1H, OH), 8.49 (t, 1H, *J* 5.6 Hz). ^{13}C NMR (151 MHz, DMSO-d6) δ = 11.8, 18.2, 20.4, 23.2, 23.8, 26.1, 26.9, 27.7, 30.4, 31.2, 31.8, 34.2, 34.8, 35.1, 35.4, 36.3, 41.5, 42.2, 55.5, 50.1, 69.8, 117.7 (C≡N), 173.2 (CONH). HRMS (ESI) calcd for $C_{26}H_{42}N_2O_2$: 437.3144; found: 437.3138 [M+Na]$^+$.

Please cite this article as: Magiera et al., Lithocholic Acid Hydroxyamide Destabilizes Cyclin D1 and Induces G_0/G_1 Arrest by Inhibiting Deubiquitinase USP2a, Cell Chemical Biology (2017), http://dx.doi.org/10.1016/j.chembiol.2017.03.002

^1H NMR of compound LCACN (600 MHz, DMSO-d_6):

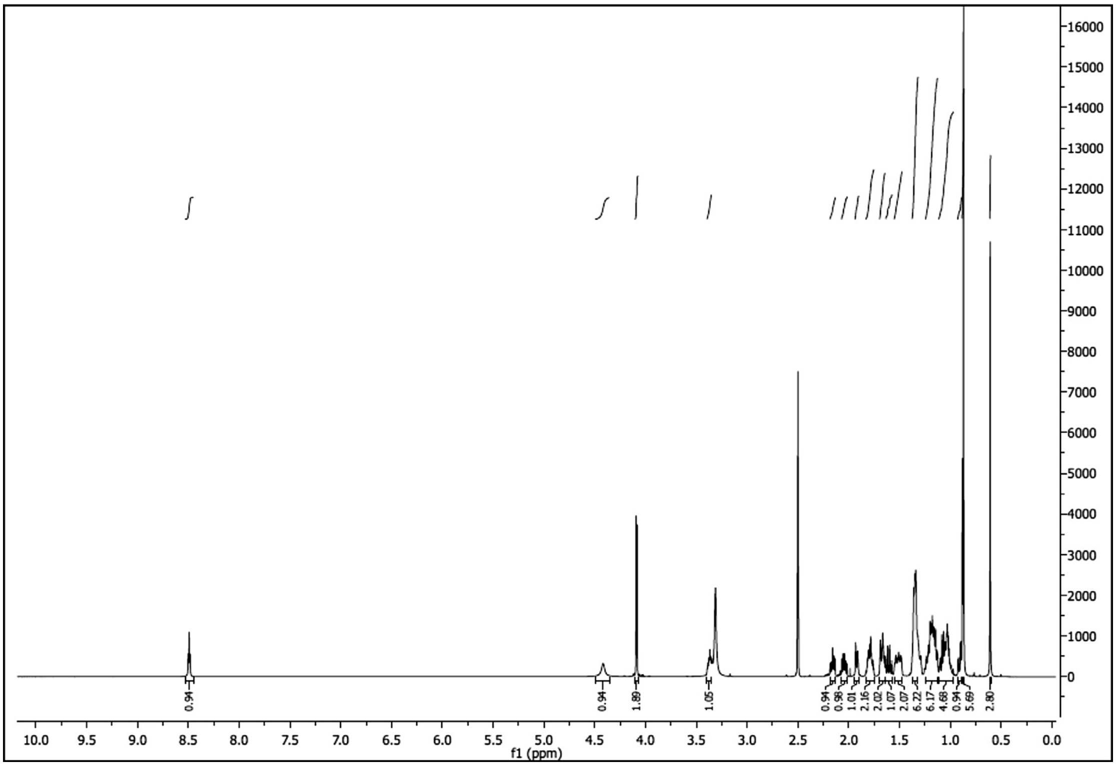

^{13}C NMR of compound LCACN (151 MHz, DMSO-d_6):

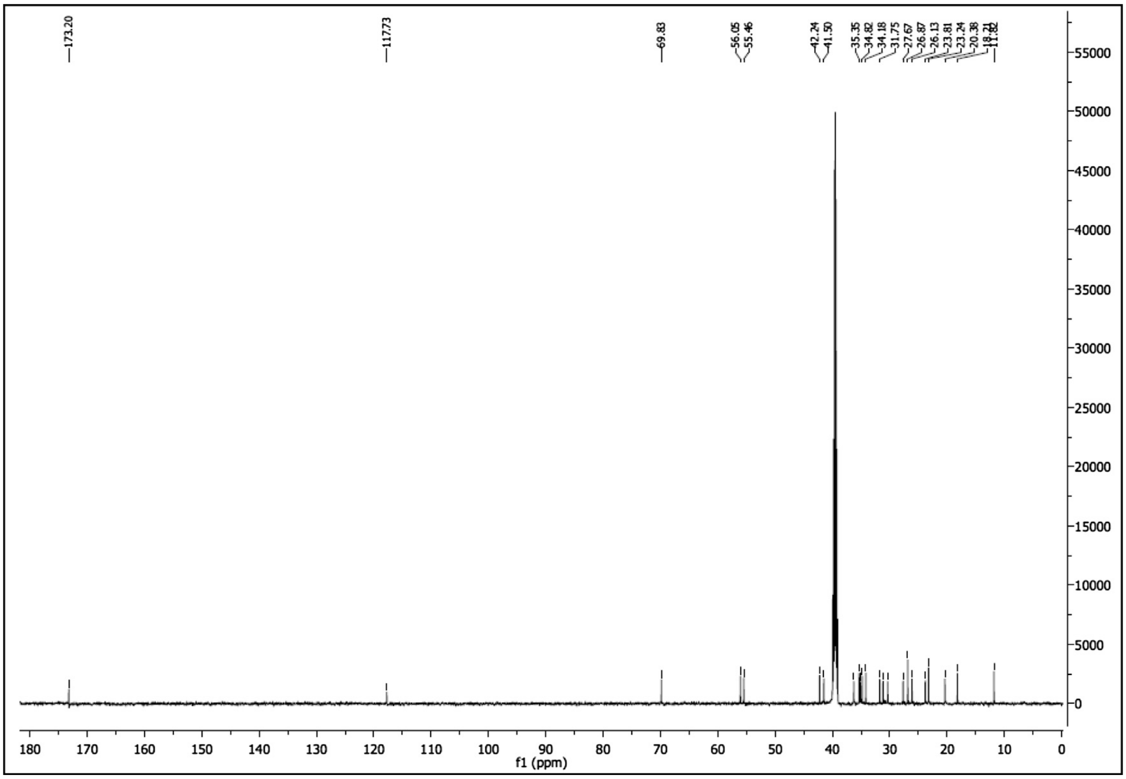

Methyl 3-Hydroxylithocholanoate (LCAME)

To the suspension of lithocholic acid (500 mg, 1.33 mmol) in dry methanol (5 ml) acetyl chloride (50 μl, 0.70 mmol) was added dropwise. The flask with reactants was sealed with septum and stirred for 4h at RT. Then distilled water (5 ml) was added resulting in white precipitate that was collected by filtration. Product was recrystallized from AcCN giving (434 mg, 1.11 mmol) of white crystals (90% yield). mp 128-132°C. IR (ATR): 3516, 3329, 2933, 2861, 1732 cm^{-1}. ^{1}H NMR (300 MHz, DMSO-d6) δ = 0.64 (s, 3H, CH_3), 0.85-0.88 (m, 6H), 0.89-1.27 (m, 11H), 1.27-1.40 (m, 7H), 1.46-1.72 (m, 5H), 1.75-1.83 (m, 2H), 1.90-1.95 (m, 1H), 2.16-2.24 (m, 1H), 2.28-2.36 (m, 1H), 3.36 (s, 1H), 3.57 (s, 3H), 4.42 (d, J 4.61 Hz, 1H). ^{13}C NMR (75 MHz, DMSO-d6) δ = 11.8, 18.8, 20.4, 23.2, 23.8, 26.1, 26.9, 27.7, 30.3, 30.6, 34.2, 34.8, 35.1, 35.4, 36.3, 41.5, 42.2, 51.2 (O$-$$CH_3$), 55.4, 56.0, 69.8, 173.7 (C=O). HRMS (ESI) calcd for $C_{25}H_{42}O_3$: 413.3032; found: 413.3023 $[M+Na]^+$.

^{1}H NMR of compound LCAME (300 MHz, DMSO-d_6):

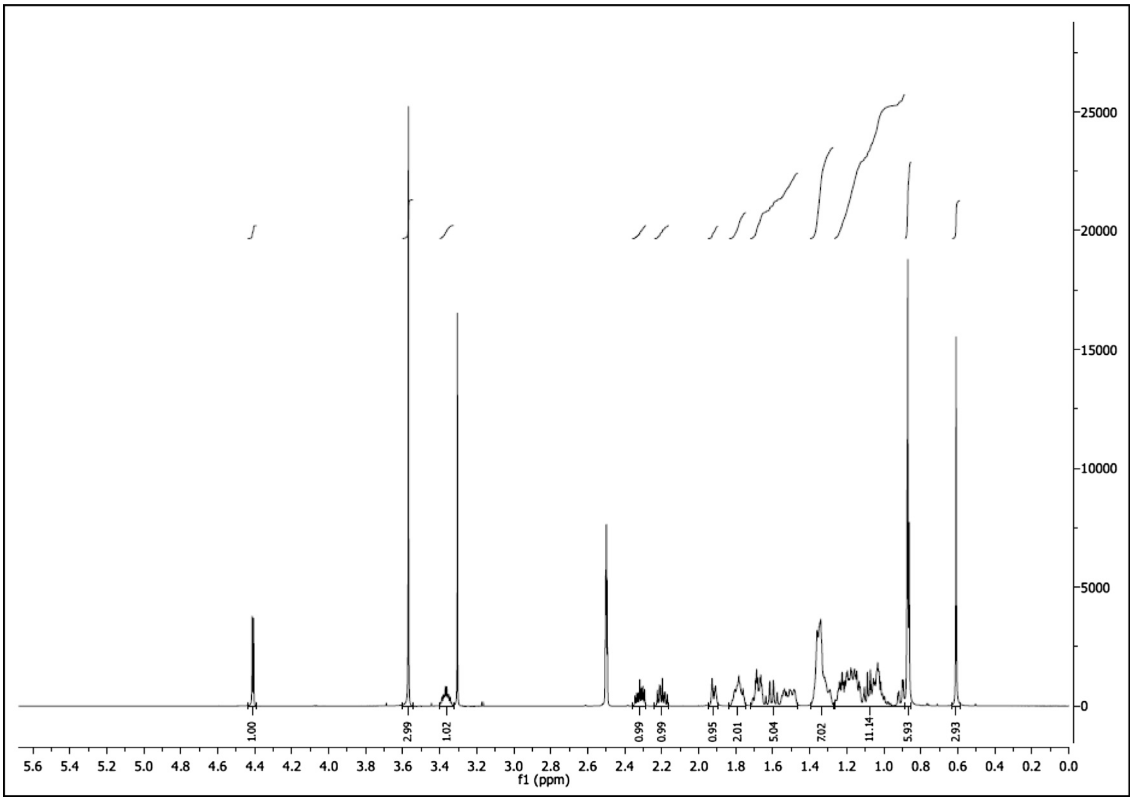

^{13}C NMR of compound LCAME (75 MHz, DMSO-d_6):

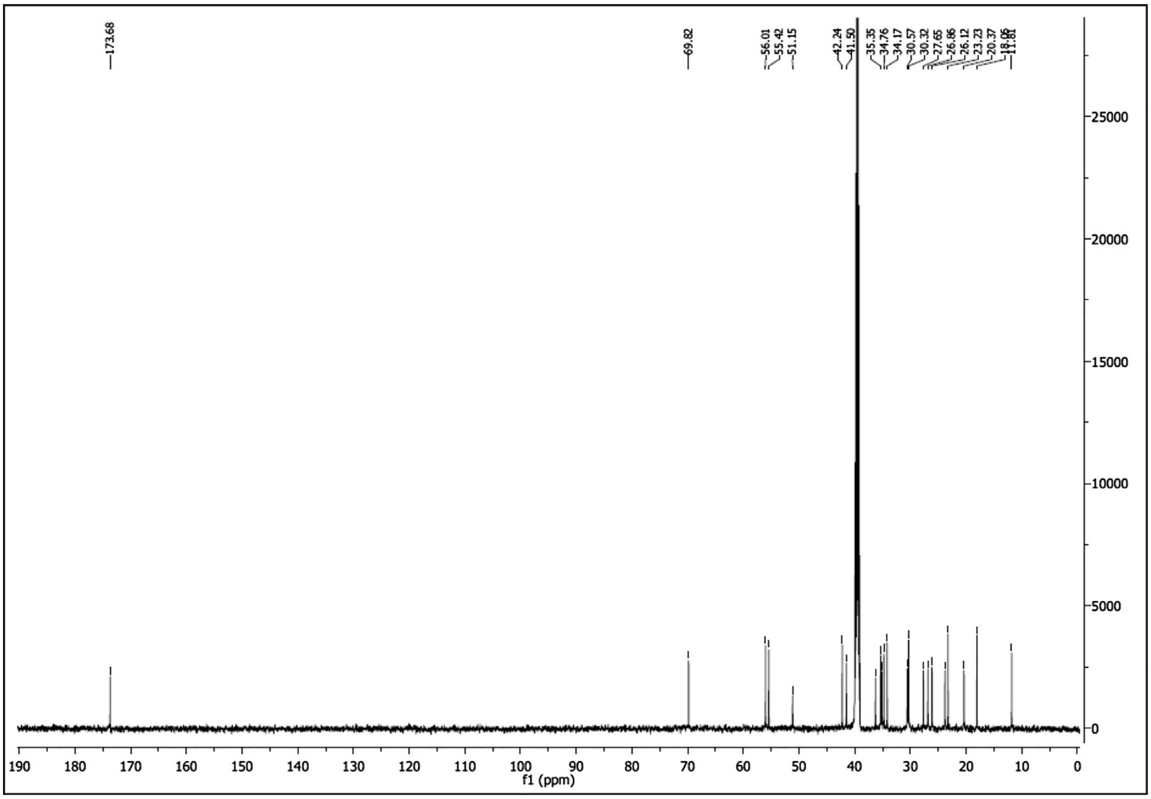

3-Oxo-Lithocholic Acid (LCAK)

CrO_3 (200 mg, 2 mmol) was suspended in 0.2 ml of conc. H_2SO_4 acid and cooled to 0°C to be diluted with 0.6 ml of cold distilled water. So obtained Jones Reagent was dropped to the cold solution (100 mg, 2.60 mmol) of lithocholic acid in 10 ml of acetone. The mixture was stirred in 0°C for an hour. Acetone was removed and the residue was diluted with 20 ml of diethyl ether to be washed with saturated solution of $NaHCO_3$ and brine. Organic layer was dried over anhydrous Na_2SO_4 and evaporated. The crude product was crystalized from methanol giving (64 mg, 0.171 mmol) of white crystals (65% yield). mp 120-123°C. IR (ATR): 2927, 2879, 1699, 1447, 1308 cm^{-1}. ^{1}H NMR (300 MHz, DMSO-d6) δ = 0.65 (s, 3H), 0.88 (d, J = 6.5 Hz, 3H), 0.96 (s, 3H), 1.31-1.01 (m, 10H), 1.47-1.34 (m, 4H), 1.62-1.49 (m, 2H), 1.88-1.64 (m, 5H), 1.95 (m, 3H), 2.43-2.03 (m, 3H), 2.74 (m, 1H), 11.88 (s, 1H). ^{13}C NMR (75 MHz, DMSO-d6) δ = 11.8, 18.1, 20.7, 23.7, 25.2, 26.2, 27.6, 30.7, 30.7, 34.4, 34.8, 35.0, 36.4, 36.7, 41.8, 42.3, 43.5, 55.6, 174.8, 211.7. HRMS (ESI) calcd for $C_{24}H_{38}O_3$: 397.2719; found: 397.2719 $[M+Na]^+$.

^1H NMR of compound LCAK (300 MHz, $CDCl_3$):

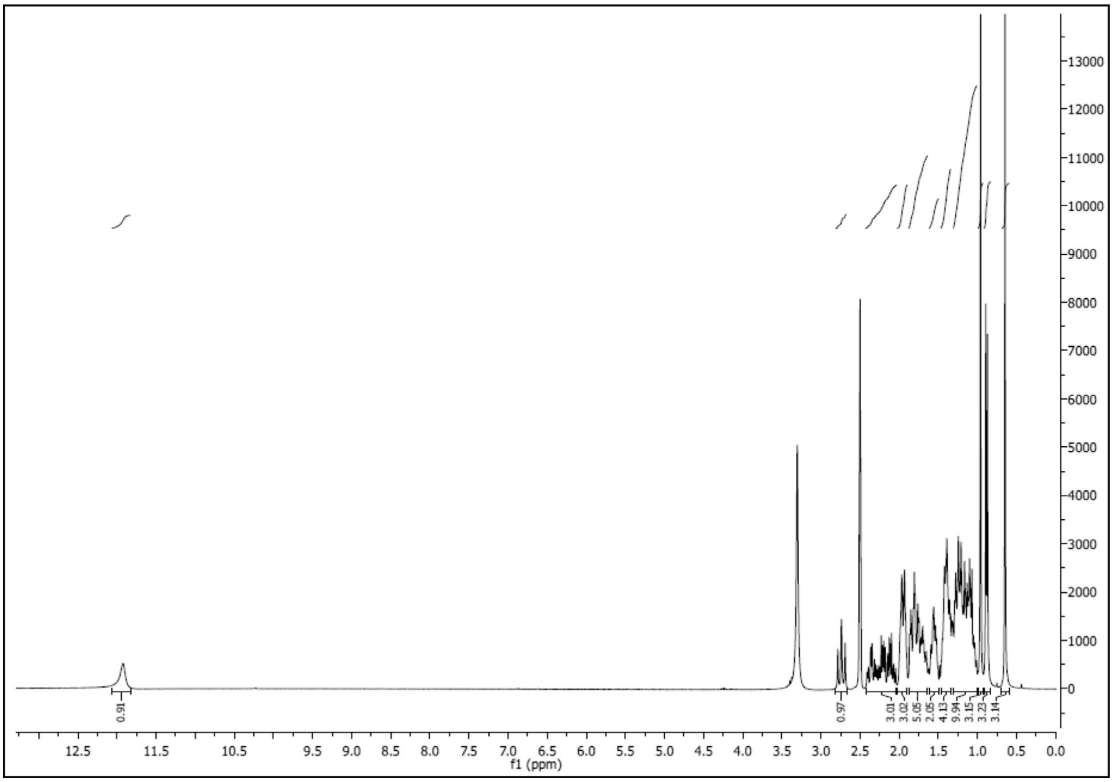

^{13}C NMR of compound LCAK (75 MHz, $CDCl_3$):

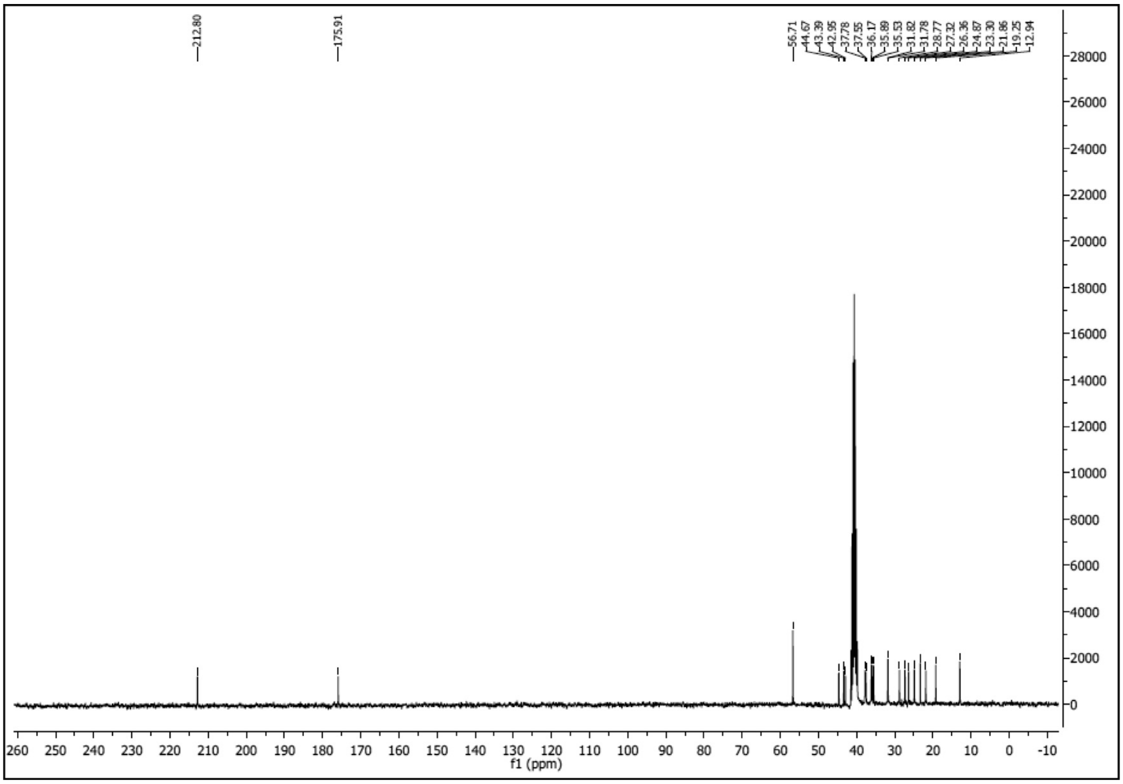

3-Acetoxy-Lithocholic Acid (LCAE)

To acetyl chloride 1 ml stirred at 0°C lithocholic acid (100 mg, 0.26 mmol) was added in portions. The mixture was removed from the ice bath and the suspension was left overnight at RT. Acetyl chloride was evaporated under reduced pressure. The residue was dried under vacuum and recrystallized from acetic acid as a white solid 81 mg (90% yield), mp 168-172°C. IR (ATR): 2934, 2867, 1732, 1707 cm^{-1}. ^{1}H NMR (600 MHz, $CDCl_3$) δ = 0.64 (s, 3H, CH_3), 0.90-0.93 (m, 6H), 1.04-1.18 (m, 5H), 1.20-1.34 (m, 6H), 1.35-1.47 (m, 6H), 1.63-1.71 (m, 1H), 1.65-1.70 (m, 1H), 1.75-1.90 (m, 5H), 1.93-1.99 (m, 1H), 2.01-2.05 (s, 3H), 2.15-2.22 (m, 1H), 2.27-2.39 (m, 1H), 4.09-4.14 (q, J 7.05 Hz, 2H), 4.68-4.75 (m, 1H). ^{13}C NMR (151 MHz, $CDCl_3$) δ = 12.2, 14.4, 18.4, 21.0, 21.6, 23.5, 24.3, 26.5, 26.8, 27.2, 28.3, 31.2, 31.5, 32.4, 34.7, 35.2, 35.5, 40.0, 40.3, 40.6, 42.1, 42.9, 56.2, 56.7, 60.3, 74.6, 170.8 (C=O), 174.5 (C=O). HRMS (ESI) calcd for $C_{26}H_{42}O_4$: 441.2981; found: 441.2975 $[M+Na]^+$.

^{1}H NMR of compound LCAE (600 MHz, $CDCl_3$):

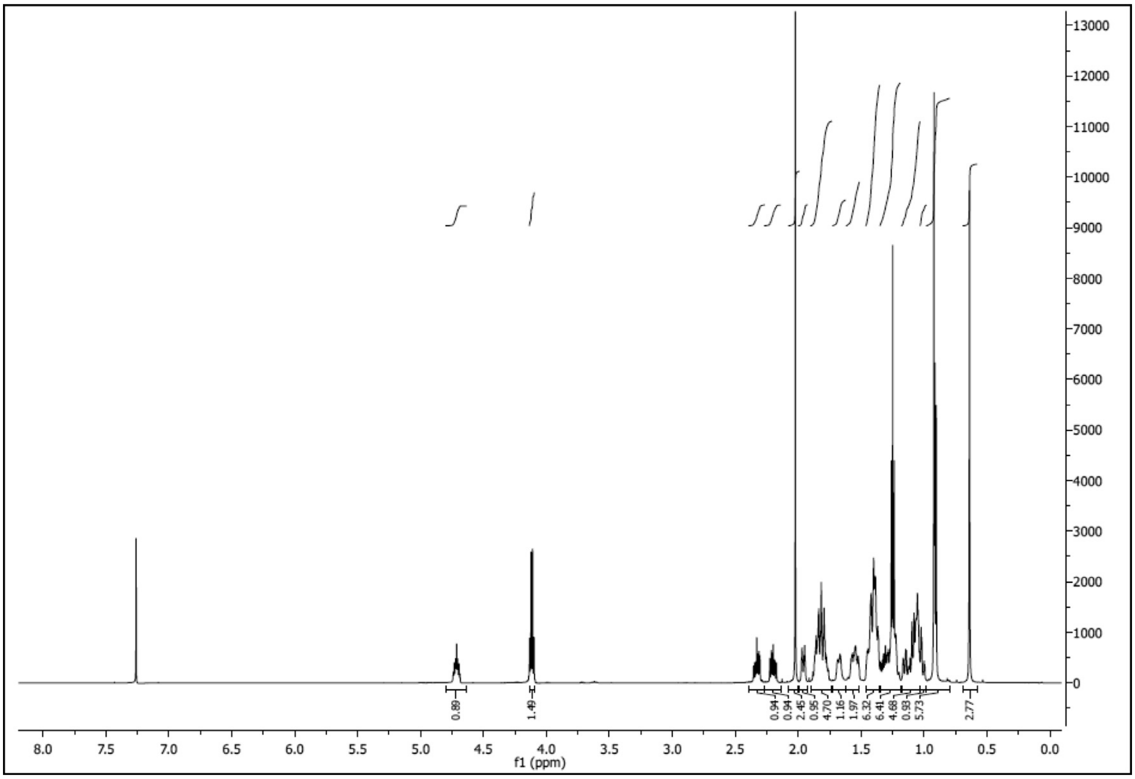

Please cite this article as: Magiera et al., Lithocholic Acid Hydroxyamide Destabilizes Cyclin D1 and Induces G_0/G_1 Arrest by Inhibiting Deubiquitinase USP2a, Cell Chemical Biology (2017), http://dx.doi.org/10.1016/j.chembiol.2017.03.002

^{13}C NMR of compound LCAE (151 MHz, $CDCl_3$):

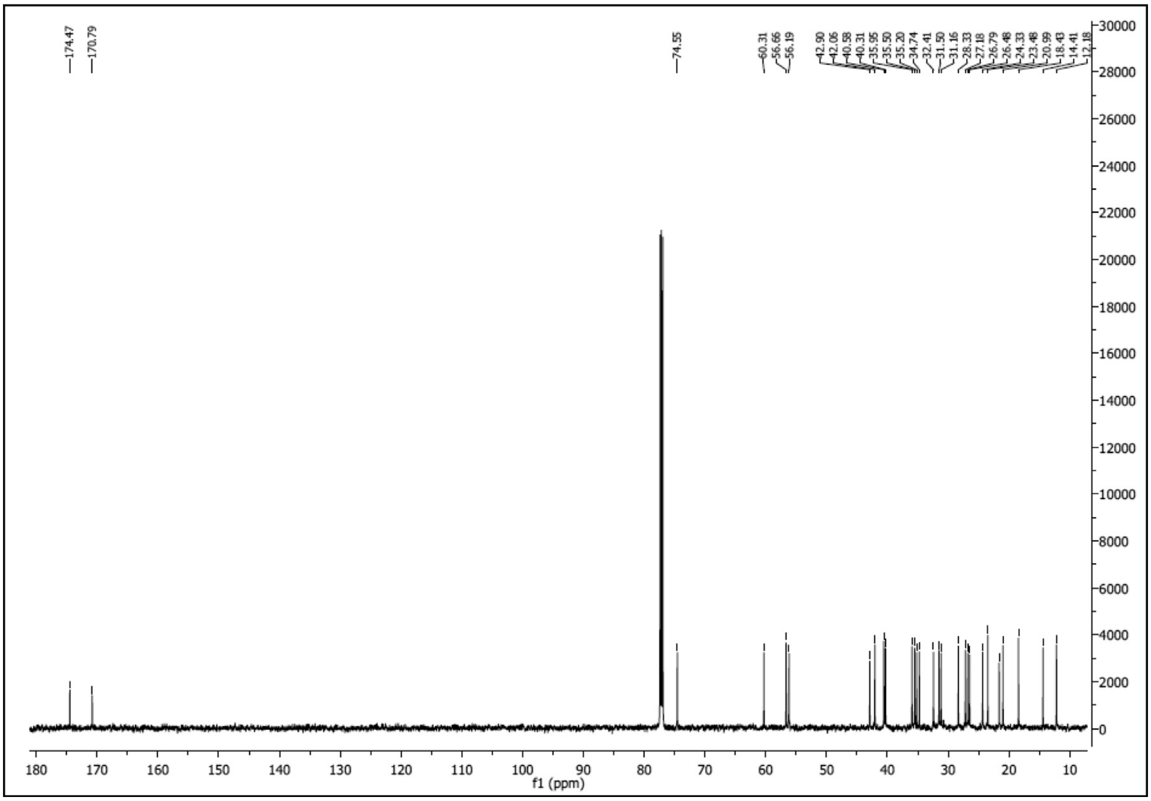

N-(hydroxy)-3-hydroxylithocholanamide (LCAHA)

Lithocholic acid (100 mg, 0.26 mmol) and 17 μl of 50% water solution of (9 mg, 0.26mmol) hydroxylamine was treated according to the synthesis of 3-hydroxylithocholanamide. The crude product was crystalized from methanol as white needles (68 mg, 0.17mmol) (65 % yield). mp 186-190°C. IR (ATR): 3358, 3181, 2940, 2853, 1652, 1471, 1445 cm^{-1}. ^{1}H NMR (300 MHz, DMSO-d6) δ= 0.61 (s, 3H), 0.90-0.85 (m, 6H), 1.26-0.92 (m, 11H), 1.43-1.26 (m, 7H), 1.57-1.44 (m, 2H), 1.72-1.57 (m, 3H), 1.87- 1.72 (m, 3H), 2.05-1.88 (m, 2H), 3.44-3.33 (m, 1H), 4.41 (d, J = 4.5 Hz, 1H), 8.61 (d, J = 1.2 Hz, 1H), 10.29 (s, 1H). ^{13}C NMR (75 MHz, DMSO-d6) δ= 11.9, 18.2, 20.4, 23.2, 23.8, 26.1, 26.9, 27.7, 29.2, 30.4, 31.4, 34.2, 34.8, 35.1, 35.4, 36.3, 39.7, 40.0, 41.5, 42.2, 48.6, 55.5, 56.1, 69.8, 169.5. HRMS (ESI) calcd for $C_{24}H_{41}NO_3$: 414.2984; found: 414.2984 $[M+Na]^+$.

^{1}H NMR of compound LCAHA (300 MHz, DMSO-d_6):

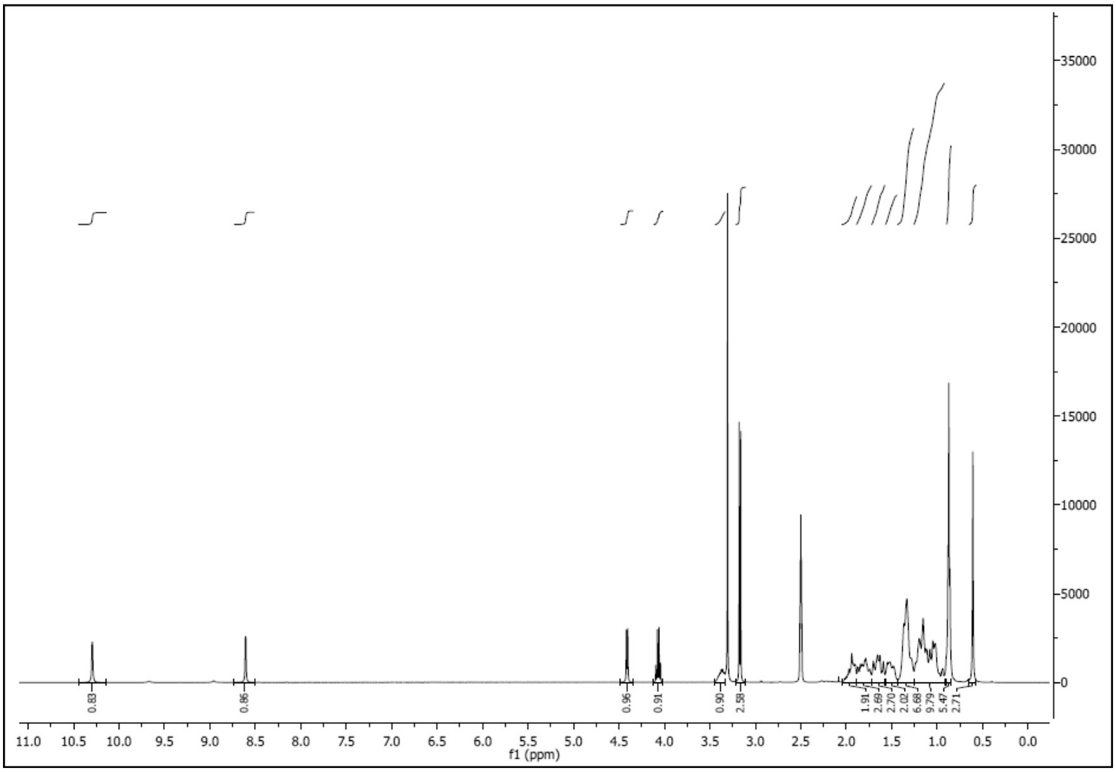

^{13}C NMR of compound LCAHA (75 MHz, DMSO-d_6):

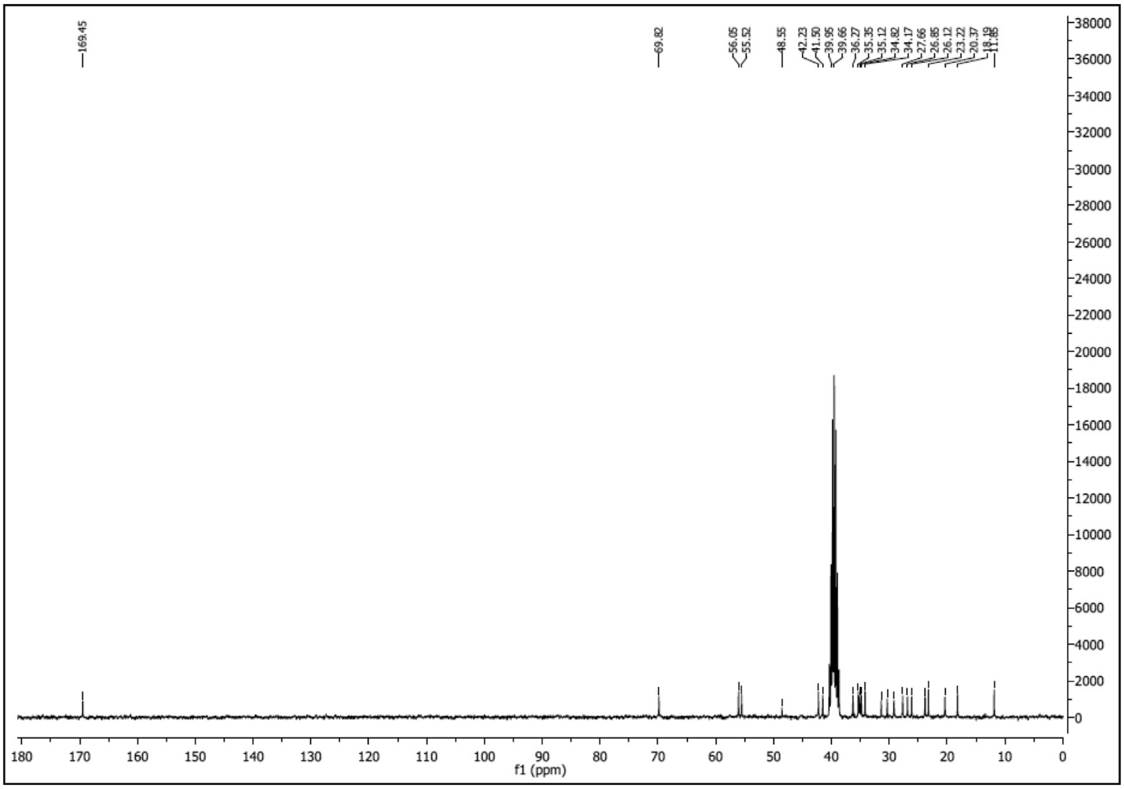

QUANTIFICATION AND STATISTICAL ANALYSIS

For the comparison of three or more groups, one-way Analysis of Variance (ANOVA) with Tukey's post-hoc test was used for the testing of significance level. For the comparison of two groups, t test was performed.

Statistical values including the exact n and statistical significance are reported in the Figure Legends.

DATA AND SOFTWARE AVAILABILITY

N/A.

ADDITIONAL RESOURCES

N/A.

Cell Chemical Biology, Volume *24*

Supplemental Information

Lithocholic Acid Hydroxyamide Destabilizes Cyclin D1 and Induces G_0/G_1 Arrest by Inhibiting Deubiquitinase USP2a

Katarzyna Magiera, Marcin Tomala, Katarzyna Kubica, Virginia De Cesare, Matthias Trost, Bartosz J. Zieba, Neli Kachamakova-Trojanowska, Marcin Les, Grzegorz Dubin, Tad A. Holak, and Lukasz Skalniak

Supplemental Figures

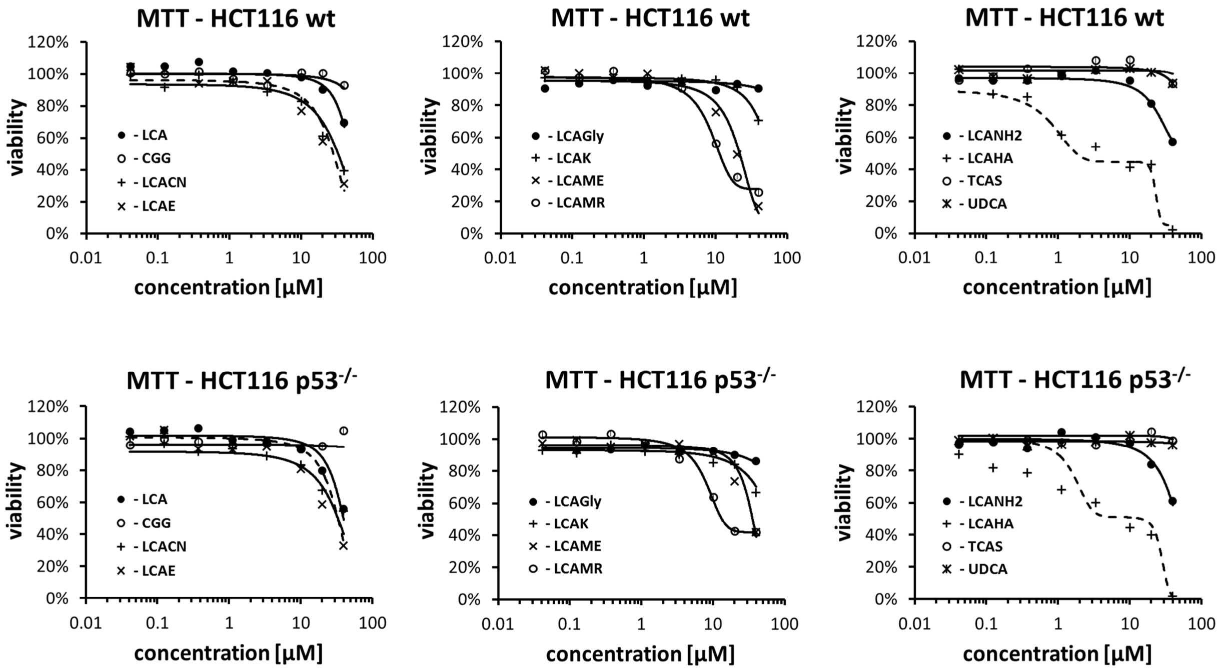

Figure S1. The impact of LCA and its derivatives on the growth and survival of HCT116 p53^{wt} and HCT116 p53$^{-/-}$ cell lines. Related to Figure 1 and Table 2.

For MTT assay, the cells were seeded at low confluence and treated with indicated concentrations of the compounds for six days. The graphs show representative results from three experiments.

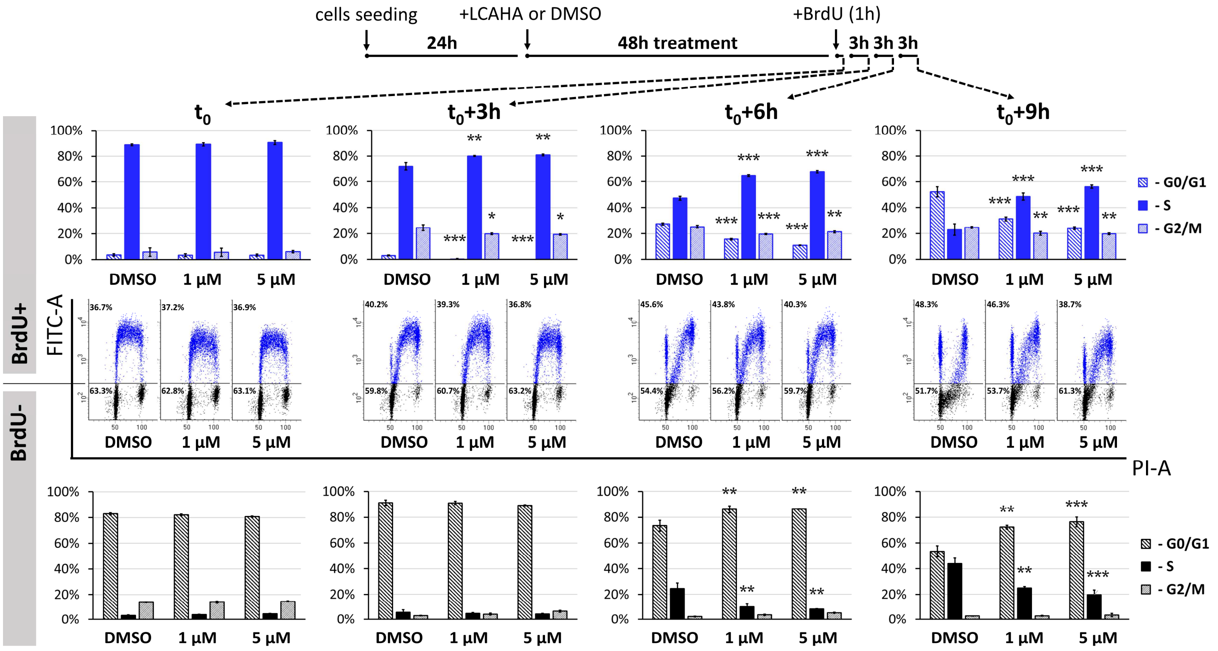

Figure S2. PI-BrdU pulse-chase labeling of HCT116 cells for the evaluation of cell cycle progression following LCAHA treatment. Related to Figure 1.

HCT116 p53[wt] cells were seeded on culture dishes in growth medium containing 3.3% FBS and treated with LCAHA in a concentration of 1 µM or 5 µM for 48 hours, or with DMSO as a control. One hour before the end of the treatment half of the medium was collected in a falcon tube, and the other half was supplemented with BrdU in a final concentration of 10 µM. After one hour, the medium containing BrdU was removed and the cells were trypsinized and fixed (time "t_0") or cultured in the medium previously collected in the falcon tube for additional 3, 6 or 9 hours. After that time the cells were trypsinized, fixed and stained with FITC conjugated anti-BrdU antibody and propidium iodide (PI). The cells were analyzed by flow cytometry using BD Fortessa cell analyzer and BD FACSDIVA software. All cells, as well as FITC positive (BrdU+, top panel) and FITC negative (BrdU-, bottom panel) counts were exported to separate files and analyzed separately using ModFit LT Software. The graphs show mean ± SD values from three independent experiments. The statistical significance was evaluated using one-way ANOVA with Tukey's post-hoc test: * $p<0.05$, ** $p<0.01$, *** $p<0.001$.

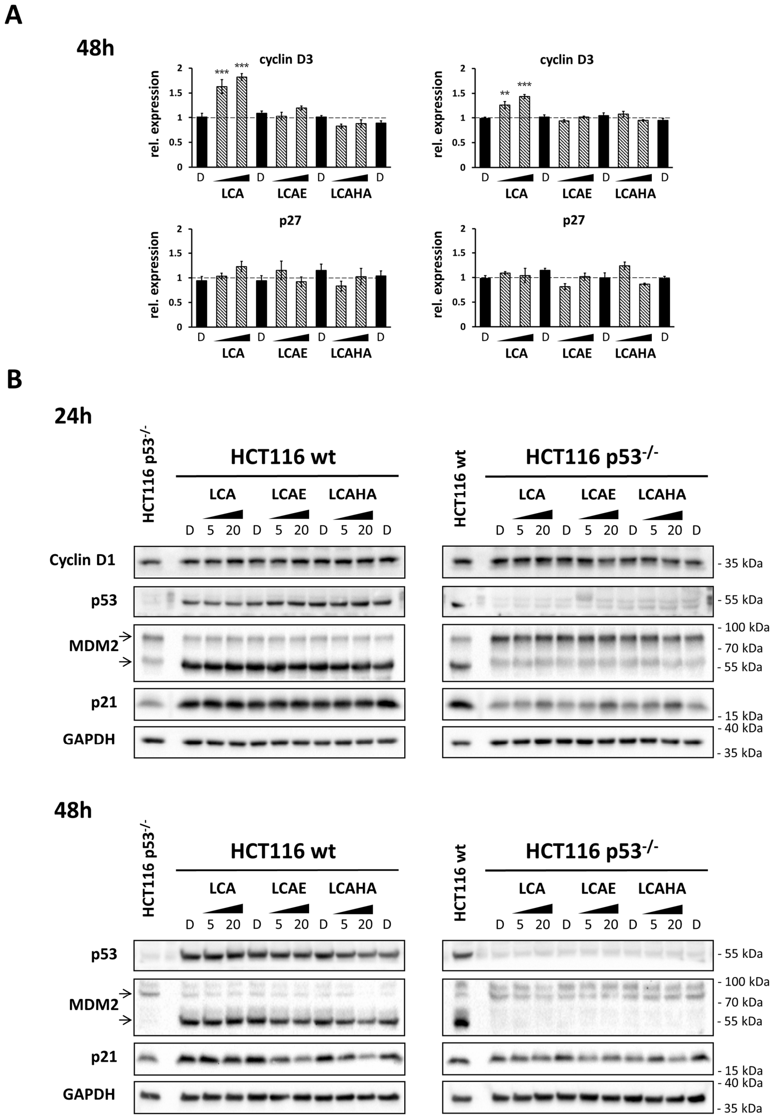

Figure S3. The analysis of the expression of cyclin D3 and p27 and the activation of p53 pathway by LCA, LCAE and LCAHA. Related to Figure 2.

(A) The graphs present densitometry analysis of cyclin D3 and p27 expression form western blot results presented in the Figure 2A of the manuscript. The graphs show mean ± SEM from three independent experiments. The statistical significance was evaluated using one-way ANOVA with Tukey's post-hoc test: ** $p<0.01$, *** $p<0.001$.

(B) HCT116 $p53^{wt}$ (left panels) or HCT116 $p53^{-/-}$ (right panels) cells were treated with the indicated concentrations of the compounds for 24 (top) or 48 (bottom) hours. Western blot analysis was performed with the indicated antibodies. The figure shows western blot images representative of three independent experiments.

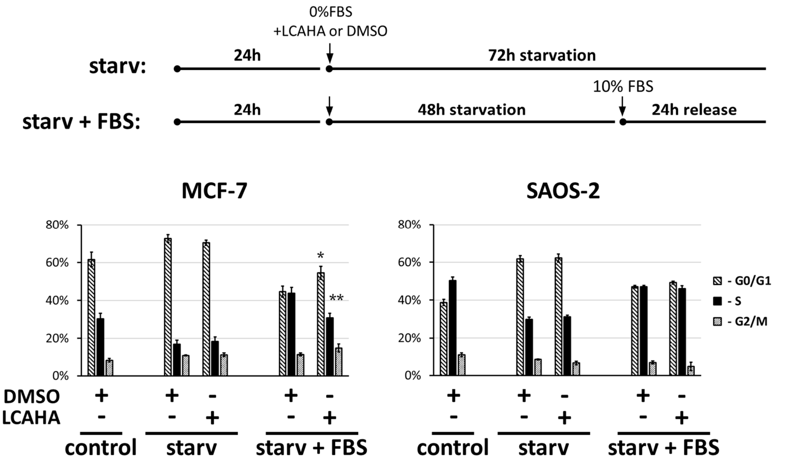

Figure S4. The analysis of the progression of MCF-7 and SAOS-2 cells through the cell cycle following serum starvation and release by the addition of serum. Related to Figure 3.

For the experiment, cyclin D1-dependent MCF-7 and cyclin D1-negative SAOS-2 cell lines were used. The cells were synchronized in G0/G1 by the removal of FBS for 2 days of the culture in the presence of LCAHA compound. After this time, the cells were released by the addition of the medium supplemented with 10% FBS for 24 hours (still in the presence of the compound). The cells were fixed, stained with PI and analysed for cell cycle distribution with ModFit LT Software. The graphs show mean ± SD values from three independent experiments. The statistical significance was evaluated using t-test: * $p<0.05$, ** $p<0.01$.

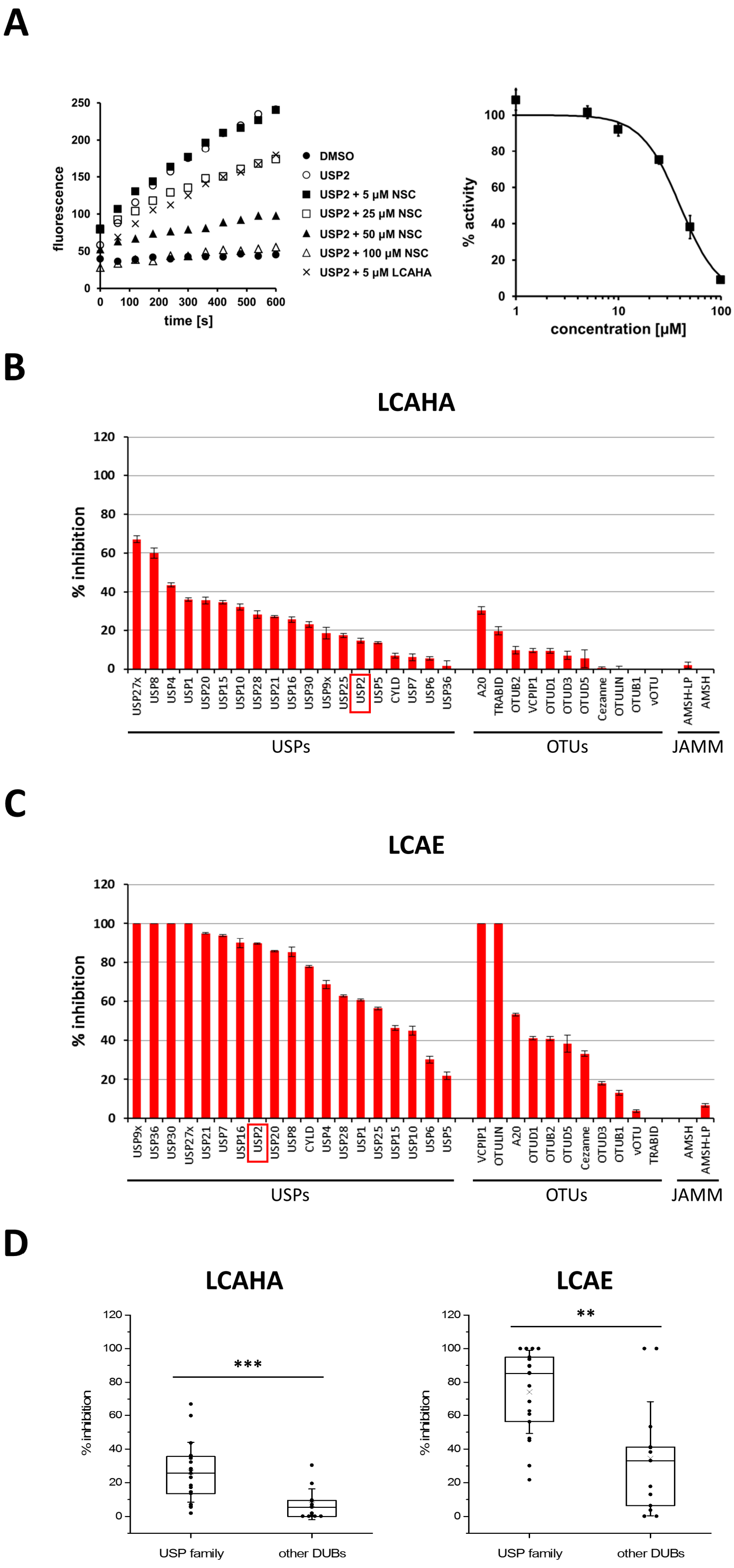

A
fluorescence
time [s]
DMSO
USP2
USP2 + 5 µM NSC
USP2 + 25 µM NSC
USP2 + 50 µM NSC
USP2 + 100 µM NSC
USP2 + 5 µM LCAHA
% activity
concentration [µM]
B
LCAHA
% inhibition
USP27x USP8 USP4 USP1 USP20 USP15 USP10 USP28 USP21 USP16 USP30 USP9x USP25 USP2 USP5 CYLD USP7 USP6 USP36
A20 TRABID OTUB2 VCPIP1 OTUD1 OTUD3 OTUD5 Cezanne OTULIN OTUB1 vOTU
AMSH-LP AMSH
USPs
OTUs
JAMM
C
LCAE
% inhibition
USP9x USP36 USP30 USP27x USP21 USP7 USP16 USP2 USP20 USP8 CYLD USP4 USP28 USP1 USP25 USP15 USP10 USP6 USP5
VCPIP1 OTULIN A20 OTUD1 OTUB2 OTUD5 Cezanne OTUD3 OTUB1 vOTU TRABID
AMSH AMSH-LP
USPs
OTUs
JAMM
D
LCAHA
LCAE
% inhibition
***
**
USP family
other DUBs

Figure S5. The analysis of the activity of NSC 632839 and the selectivity of LCAHA and LCAE compounds. Related to Figure 5.

(A) The effects of the NSC 632839 compound (inhibitor control) on the USP2a activity tested using the Ub-AMC hydrolysis assay. Left panel: The Ub-AMC hydrolysis assay demonstrates that 25 µM NSC 632839 inhibits the USP2a enzymatic activity at a similar level as 5 µM LCAHA compound. The graph shows representative data of multiple repeats. Right panel: A dose-response inhibition of USP2a activity measured in the Ub-AMC hydrolysis assay, in the presence of various concentrations of the NSC 632839 compound. The graph shows the mean ± SD values from the 3 experiments.
(B-D) High throughput MS-based enzyme activity assay was performed on a panel of DUB enzymes in a presence of 100 µM LCAHA (B) or LCAE (C) compounds. The graphs present mean ± SD values from duplicates. (D) The comparison of the inhibitory potential of the tested compounds towards USP proteins and other DUB enzymes. The box charts present 25/75th percentiles ± SD values. All data points are also presented on the chart. The statistical significance was calculated with U Mann-Whitney test: ** $p<0.01$, *** $p<0.001$.

Supplemental Tables

Kinetic constants	Lineweaver-Burk equation		
	USP2a	USP2a + 2 μM LCAHA	USP2a + 5 μM LCAHA
K_m [μM]	12.2 ± 2.8	12.1 ± 1.8	12.6 ± 3.0
V_{max} [μMs^{-1}]	$1.4 \cdot 10^{-2} \pm 2 \cdot 10^{-3}$	$9.9 \cdot 10^{-3} \pm 9.7 \cdot 10^{-4}$	$3.8 \cdot 10^{-3} \pm 1.3 \cdot 10^{-3}$

Table S1. The kinetic constants for USP2a in the presence of the LCAHA compound. Related to Figure 5.

Values are determined based on the kinetic analysis of USP2a-catalyzed Ub-AMC hydrolysis. Presented data are determined by fitting the Lineweaver-Burk equation.
